# Advanced Microscopy Reveals Complex Developmental and Subcellular Localization Patterns of ANNEXIN 1 in *Arabidopsis*

**DOI:** 10.3389/fpls.2020.01153

**Published:** 2020-08-05

**Authors:** Michaela Tichá, Hendrik Richter, Miroslav Ovečka, Nicola Maghelli, Miroslava Hrbáčková, Petr Dvořák, Jozef Šamaj, Olga Šamajová

**Affiliations:** ^1^ Centre of the Region Haná for Biotechnological and Agricultural Research, Faculty of Science, Palacký University Olomouc, Olomouc, Czechia; ^2^ Institute of Celullar and Molecular Botany, University of Bonn, Bonn, Germany; ^3^ Max Planck Institute of Molecular Cell Biology and Genetics, Advanced Imaging Facility, Dresden, Germany

**Keywords:** annexin 1, *Arabidopsis thaliana*, cell division, development, lattice light-sheet fluorescence microscopy, light-sheet fluorescence microscopy, plasma membrane, subcellular localization

## Abstract

Annexin 1 (ANN1) is the most abundant member of the evolutionary conserved multigene protein superfamily of annexins in plants. Generally, annexins participate in diverse cellular processes, such as cell growth, differentiation, vesicle trafficking, and stress responses. The expression of annexins is developmentally regulated, and it is sensitive to the external environment. *ANN1* is expressed in almost all *Arabidopsis* tissues, while the most abundant is in the root, root hairs, and in the hypocotyl epidermal cells. Annexins were also occasionally proposed to associate with cytoskeleton and vesicles, but they were never developmentally localized at the subcellular level in diverse plant tissues and organs. Using advanced light-sheet fluorescence microscopy (LSFM), we followed the developmental and subcellular localization of GFP-tagged ANN1 in post-embryonic *Arabidopsis* organs. By contrast to conventional microscopy, LSFM allowed long-term imaging of ANN1-GFP in *Arabidopsis* plants at near-environmental conditions without affecting plant viability. We studied developmental regulation of ANN1-GFP expression and localization in growing *Arabidopsis* roots: strong accumulation was found in the root cap and epidermal cells (preferentially in elongating trichoblasts), but it was depleted in dividing cells localized in deeper layers of the root meristem. During root hair development, ANN1-GFP accumulated at the tips of emerging and growing root hairs, which was accompanied by decreased abundance in the trichoblasts. In aerial plant parts, ANN1-GFP was localized mainly in the cortical cytoplasm of trichomes and epidermal cells of hypocotyls, cotyledons, true leaves, and their petioles. At the subcellular level, ANN1-GFP was enriched at the plasma membrane (PM) and vesicles of non-dividing cells and in mitotic and cytokinetic microtubular arrays of dividing cells. Additionally, an independent immunolocalization method confirmed ANN1-GFP association with mitotic and cytokinetic microtubules (PPBs and phragmoplasts) in dividing cells of the lateral root cap. Lattice LSFM revealed subcellular accumulation of ANN1-GFP around the nuclear envelope of elongating trichoblasts. Massive relocation and accumulation of ANN1-GFP at the PM and in Hechtian strands and reticulum in plasmolyzed cells suggest a possible osmoprotective role of ANN1-GFP during plasmolysis/deplasmolysis cycle. This study shows complex developmental and subcellular localization patterns of ANN1 in living *Arabidopsis* plants.

## Introduction

Plants as sessile organisms cannot easily abandon the unfavorable environment. Thus, they developed sophisticated molecular and genetic mechanisms for signal perception and transduction helping them to adapt and survive. These mechanisms include annexins that can integrate several developmental and cellular functions and participate in plant stress responses.

Annexins represent an evolutionary highly conserved superfamily of phospholipid-binding proteins, abundant in all eukaryotes (Lizarbe et al., 2013). This multifunctional group of proteins is involved in the coordination of fundamental processes, such as cell growth, differentiation, and stress responses (Clark et al., 2012; Yadav et al., 2018; He et al., 2020; Saad et al., 2020). Annexins can likely play roles in intracellular vesicular trafficking (endocytosis and exocytosis) and bind to cytoskeletal components and membrane phospholipids depending on Ca^2+^ ions. Based on the structure analysis (Hofmann et al., 2000), annexins have been proposed as atypical membrane channels, because they lack transmembrane domains characteristic for Ca^2+^ permeable channel proteins (Gerke and Moss, 2002; White et al., 2002). Nevertheless, some experiments confirmed that *ANN1* gene encodes the hyperpolarization-activated Ca^2+^ channel (Véry and Davies, 2000).

Plant annexins, unlike animal ones, form a distinct monophyletic group (Mortimer et al., 2008). The primary amino acid sequences of plant annexins are considerably different; however, the overall structure is preserved within four recognizable repeated domains (I-IV) of about 70 amino acids called C-terminal core (Moss and Morgan, 2004). Every domain contains four alpha helices (A, B, D, and E), while a fifth alpha helix (C) is arranged almost perpendicular to other ones (Gerke and Moss, 2002). Annexins possess a special sequence known as “endonexin” fold, which is responsible for calcium binding (Hofmann et al., 2003). Variation of the primary sequence of core domains is important for the functional divergence of plant annexins (Clark et al., 2012). A single annexin molecule resembles a slightly curved disk. The convex surface includes calcium and phospholipid-binding sites (Mortimer et al., 2008). The more concave surface faces the cytoplasm and interacts with NH_2_-terminal domains of proteins or other cytoplasmic molecules. Depending on the concentration of Ca^2+^, pH, lipid composition, and voltage annexins can associate with intracellular membranes or be inserted to them (Gerke and Moss, 2002).

The genome of *Arabidopsis thaliana* comprises eight different annexin genes *ANN1*–*ANN8* (Clark et al., 2001) that encode proteins of molecular mass between 32 and 42 kDa. *ANN1* is located on chromosome 1, *ANN3* and *ANN4* are on chromosome 2, and *ANN6* and *ANN7* are present on chromosome 5 in a tandem arrangement. Generally, the primary sequences of individual plant annexin genes are rather different. The highest similarity was found between *ANN2*, *ANN6*, and *ANN7* with approximately 76–83% identity at the deduced amino acid level (Cantero et al., 2006).

The ability to bind negatively charged phospholipids in a calcium-dependent manner is a typical feature of all annexins. They associate with membrane lipids such as phosphatidylserine, phosphatidylglycerol, and phosphatidylinositol, as well as with phosphatidic acid, whereas different annexins may differ in their specificity to various phospholipids and sensitivity to Ca^2+^ (Gerke and Moss, 2002). The calcium-binding site of type II comprises GXGTD sequence within highly conserved endonexin fold (Clark et al., 2001). The cytosolic free calcium concentrations ([Ca^2+^]cyt) range from 100 to 200 nM and could increase due to the signals such as light, hormones, gravity, wind, and mechanical stimuli (Clark and Roux, 1995). Eventually, annexins interact with membrane phospholipids at micromolar concentrations of Ca^2+^ in the cytoplasm. The maintenance of nanomolar free calcium concentrations is provided by Ca^2+^-sensors, Ca^2+^-binding proteins, and Ca^2+^-transporters/pumps. Annexins represent a group of proteins binding Ca^2+^ without EF-hand motif (Tuteja, 2009).

Except for Ca^2+^-binding sites, other sequences have been proposed to be important for the functional properties of annexins. Inherent peroxidase activity was originally suggested for AtANN1 (Gorecka et al., 2005; Laohavisit and Davies, 2009) based on sequence similarity with heme peroxidases comprising of 30 amino acid binding hem sequence (Gidrol et al., 1996). Other potentially important sequences are the GTP-binding motif (marked GXXXXGKT and DXXG) and the IRI motif responsible for the association with F-actin (Clark et al., 2001). Apparently, plant annexins contain protein domains important for regulation of secretion or binding to F-actin, GTP, calcium, and plasma membrane (Konopka-Postupolska, 2007; Lizarbe et al., 2013). Plant annexins are also essential for signal transduction during plant growth and development (Surpin et al., 2003), ion homeostasis (Pittman, 2012), salt and drought stress tolerance (Zhu et al., 2002; Hamaji et al., 2009; He et al., 2020), or plant defense (Leborgne-Castel and Bouhidel, 2014; Zhao et al., 2019). Experiments using polyclonal annexin antibody in corn and pea provided evidence that annexins can mediate secretion of cell wall materials during plant growth and development (Clark et al., 1994; Carroll et al., 1998). A recent study suggests new roles of ANN1 and ANN2 in post-phloem sugar transport to the root tip of *Arabidopsis* (Wang et al., 2018).

In addition, annexins also associate with mitogen activated protein kinases (MAPKs) and might participate in calcium-dependent MAPK signaling (Baucher et al., 2012). Rice annexin Os01g64970, a homolog of *Arabidopsis* ANN4, interacted with 23 kinases, participating in calcium-dependent MAPK signaling, including receptor-like kinases, Ste20 (Sterile 20-like) kinase, SPK3-kinase, and casein kinase (Rohila et al., 2006). Furthermore, the calcium-dependent interaction of ANN1 and ANN4 is important for salt and drought stress responses in *Arabidopsis* (Huh et al., 2010).

Gene expression of plant annexins is influenced by environmental and developmental signals. For example, *AtANN1* mRNA levels are up-regulated in leaves not only by application of NaCl, H_2_O_2_, drought, and wounding but also by stress hormones abscisic acid and salicylic acid (Konopka-Postupolska et al., 2009). AtANN1, the most abundant member of the family, shows different expression levels in almost all plant tissues, and the most abundant is in the root, hypocotyl epidermal cells, and in the root hairs. So far, localization of annexins was studied in fixed samples from *Arabidopsis* seedlings (Clark et al., 2001; Clark et al., 2005a; Clark et al., 2005b). Immunolocalization of ANN1 and ANN2 in *Arabidopsis* was combined with autoradiography of newly synthesized cell wall polysaccharides. A comparison of these two analyses revealed that the localization of annexins is spatio-temporally associated with the secretion of cell wall material (Clark et al., 2005a; Clark et al., 2005b). De Carvalho-Niebel et al. (2002) showed cytosolic localization of ANN1-GFP in *Medicago truncatula* roots. Several hypotheses have been proposed regarding AtANN1 localization, but a systematic study on developmental and subcellular localization patterns in living *Arabidopsis* plants was not performed. Such developmental spatio-temporal analysis of ANN1 localization at organ, tissue, and subcellular levels could help to better understand its role during post-embryonic seedling development. For this purpose, advanced light-sheet fluorescence microscopy (LSFM) provides an ideal tool to perform time-lapse 3-D imaging of deeper root tissues and structures in living *Arabidopsis* seedlings with minimal phototoxicity and photobleaching at nearly physiological conditions (Ovečka et al., 2015; Ovečka et al., 2018). Using long-term LSFM imaging, we were able to track endogenous ANN1 localization in different developmental zones, tissues, and cell types of primary and lateral roots of *Arabidopsis* transgenic line carrying *proANN1::ANN1:GFP* construct. This developmental study revealed that ANN1-GFP is an ideal molecular marker for root cap development in the primary and lateral roots of *Arabidopsis*. In addition, higher resolution of confocal laser scanning microscopy (CLSM) and Airyscan CLSM were used to monitor the overall distribution of ANN1 in aerial seedling organs and subcellular localization of ANN1 in dividing root and leaf petiole cells. Considering the complexity of developing roots, we studied the spatial distribution of ANN1-GFP using 3-D rendering of post-processed data obtained by commercial microscopy platform Luxendo MuVI SPIM. Finally, we show subcellular localization of ANN1-GFP around nuclei of trichoblast cells using lattice LSFM.

## Materials and Methods

### Preparation of Transgenic Lines Carrying ANN1-GFP

Genomic DNA was isolated from *Arabidopsis thaliana* (L.) Heynh. ecotype Columbia (Col-0) and used for amplification of the gene *AtANN1* (At1g35720.1) with putative native promoter region (altogether 2518 bp). Vector *pGwp* was created from *pCatGFP* (Reichel et al., 1996) using flanking primers to amplify from *pCatGFP* vector the GFP sequence together with the multicloning site (MCS) excluding *35S* promoter region. The primers are described in **Supplementary Table 1**. Subsequently, the amplified *pGwp* was digested with NotI and NcoI restriction enzymes, and *ANN1* promoter with *AtANN1* genomic region digested with NotI/NcoI was cloned into *pGwp* creating a fusion of *proANN1::AtANN1* with *GFP* reporter gene. The prepared cassette *proANN1::ANN1:GFP* was digested with SbfI restriction enzyme and ligated into the binary vector *pCB302* that was digested with PstI restriction enzyme that is compatible to SbfI. The binary vector *pCB302* harboring *proANN1::ANN1:GFP* cassette was transformed into *Agrobacterium tumefaciens*, strain GV3101, by electroporation (Cangelosi et al., 1991). *Arabidopsis* plants stably expressing *proANN1::ANN1:GFP* were prepared by the floral dip method (Davis et al., 2009). Successfully transformed seedlings of the T1 generation were identified on the selection medium containing BASTA herbicide. No phenotypic defects were observed in transgenic lines in T3 generation in comparison to wild-type plants. Mutual phenotype comparison of three independent transgenic lines showed no differences, and lines were subsequently used for immunoblotting, two of them for quantitative and one for qualitative microscopic analysis.

### Plant Material and Growth Conditions

Seeds of *Arabidopsis* wild type (ecotype Columbia, Col-0), transgenic lines carrying *proANN1::ANN1:GFP* construct (*ANN1-GFP*) or *35S::sGFP* construct for free GFP cloned using pMAT037 plasmid (Matsuoka and Nakamura, 1991; Mano et al., 1999) were surface-sterilized and sown on solidified 0.5% (w/v) gellan gum half-strength MS medium (Murashige and Skoog, 1962) with 1% sucrose. After stratification at 4°C for 4 days, Petri dishes with plant material were cultivated in an environmental chamber at 21°C at 70% humidity in a 16-h light/8-h dark cycle. Illumination intensity was 150 µmol m^-2^s^-1^.

### Protein Extraction and Immunoblotting

The abundance of ANN1-GFP fusion protein was verified in *Arabidopsis* transgenic lines carrying *proANN1::ANN1:GFP* construct. Plants of wild type and transgenic line expressing *35S::sGFP* were used as a negative and positive control, respectively. Roots and aboveground parts of 10-day-old plants were homogenized separately by liquid nitrogen to the fine powder. E-buffer used for protein extraction comprised 1 mM EGTA, 50 mM HEPES (pH 7.5), 10% (v/v) glycerol, 1 mM MgCl_2_, 75 mM NaCl, 1 mM NaF, 1 mM DTT, Complete™ EDTA-free protease inhibitor (Roche, Basel, Switzerland), and PhosSTOP™ phosphatase inhibitor mixtures (Roche, Basel, Switzerland). Samples were soaked properly with buffer, incubated on ice for 30 min, and then centrifuged at 13,000 rpm at 4°C for 20 min. Extracted proteins were denaturated by 5 min boiling with 4x Laemmli buffer consisting of 62.5 mM Tris-HCl (pH 6.8), 10% (v/v) glycerol, 2% (v/v) SDS, and 300 mM 2-mercaptoethanol (final concentrations). Protein separation was performed on 12% TGX Stain-Free™ FastCast acrylamide™ gels (Bio-Rad), whereas equal protein amounts were loaded for all samples. Subsequently, proteins were transferred to polyvinylidene difluoride (PVDF) membranes (GE Healthcare, Little Chalfont, United Kingdom) in a Western Blotting transfer cell (Bio-Rad) at 100 V for 1.5 h. Membranes were blocked with 4% (w/v) low-fat dry milk and 4% (w/v) bovine serum albumin dissolved in Tris-buffered saline (TBS, 100 mM Tris-HCl, 150 mM NaCl, pH 7.4) at 4°C overnight. Membrane washing with TBS-T (TBS with 0.1% Tween 20) was followed by incubation with primary monoclonal anti-GFP antibody (Sigma-Aldrich, Merck, USA) diluted 1:1000 in TBS-T containing 1% (w/v) BSA at room temperature (RT) for 1.5 h. After washing in TBS-T, membranes were incubated 1.5 h at RT in a HRP conjugated goat anti-mouse IgG secondary antibody (Santa Cruz Biotechnology, Santa Cruz, CA, USA) diluted 1:5000. Membranes extensively washed in TBS-T were developed by incubating in Clarity Western ECL substrate (Bio-Rad) to reveal protein bands. Chemidoc MP documentation system (Bio-Rad) was used for luminescence detection.

### Sample Preparation and Light-Sheet Fluorescence Microscopy

Plant material preparation was conducted according to the protocol of Ovečka et al. (2015). Briefly, seeds of *Arabidopsis* transgenic lines stably expressing *proANN1::ANN1:GFP* construct were surface sterilized and placed on solidified half-strength MS culture medium at 4°C for 4 days. Stratified seeds were transferred to round 90 mm × 25 mm Petri dishes filled with 80 ml of growth medium (pH 5.8) solidified with 0.5% (w/v) gellan gum. The placing of seeds into small depressions at the surface of the solid medium facilitated direct gravitropic root growth. Plants were cultivated in a growth chamber at 21°C and 70% humidity under long-day conditions (16-h light/8-h dark). Illumination intensity was 150 µmol m^-2^s^-1^. Individual 3-day-old seedlings growing in an axial pattern (the root embedded in the medium and the aerial parts exposed to air) were then inserted into the fluorinated ethylene propylene (FEP) tubes with the medium surrounding the root. The block of medium containing a sample was partially pushed out of the FEP tube to facilitate imaging of the root tip surrounded by medium only without the FEP tube wall. The tubes with seedlings were removed from the plate and were transferred into a pre-tempered (22°C) observation chamber of the LSFM filled with a liquid half-strength MS medium for *Arabidopsis* growth. After 30 min stabilization of the sample, microscopy was performed using the light-sheet Z.1 fluorescence microscope (Carl Zeiss, Germany) equipped with the PCO.Edge sCMOS camera (PCO AG, Germany) with the exposure time 30 ms, Plan-Apochromat 20×/1.0 NA water immersion detection objective (Carl Zeiss, Germany), and two LSFM 10×/0.2 NA illumination objectives (Carl Zeiss, Germany). Imaging was carried out by using dual-side illumination and pivot scan mode with a light sheet thickness of 4.52 µm. GFP was excited at 488 nm and detected between 505 and 545 nm. Images were acquired in three subsequent views aligned to each other in root growth direction (along the y coordinate) in time points of every 5 min in Z-stack mode for 7–15 h. Images scaling x, y, z was adjusted to 0.228 μm × 0.228 μm × 0.477 μm. The brightness and contrast of all images were uniformly corrected right before export and analysis.

The spatial distribution of ANN1-GFP in deep tissues of developing roots was studied by Luxendo MuVI SPIM (Bruker, Germany). Detection was performed *via* two Hamamatsu Orca-Flash 4.0 V3 sCMOS cameras with the exposure time 50 ms and two Olympus 20×/1.0 NA water immersion detection objectives. Double-side illumination was created by 2 Nikon CFI Plan Fluor W 10x/0.3 NA water immersion objectives. An additional magnification changer provided a total magnification of 33.3×. Image size was 2048 × 2048 pixels with a pixel size of 0.293 µm. Imaging was performed in Z-stack mode (540 slices) with 0.5-µm step size. GFP was excited at 488 nm and detected between 497 and 554 nm. Obtained four orthogonal views were processed by parallel registration (rigid transformation) and fusion in Luxendo Image Processor v2.3.0 and used for 3-D rendering in Arivis (Arivis AG, Germany).

The study of ANN1-GFP subcellular localization in trichoblasts was performed by lattice LSFM installed in Advanced Imaging Facility, MPI-CBG, Dresden, Germany. This high-resolution lattice LSFM was equipped with single-side illumination objective 28.5×/0.62 NA, single-side detection objective 25×/1.1 NA, and Orca Flash 4.0 sCMOS camera with exposure time 10 ms. Imaging was carried out at 4.4 s intervals for 272 time points at Z-stack mode and 161 slices with the step size of 0.25 µm. Images were processed by the Richardson-Lucy deconvolution algorithm and by using maximum intensity projection.

### Measurements and Statistical Analyses of Data From Light-Sheet Z.1 Imaging

Time-lapse images of developing *Arabidopsis* roots were acquired in Zen 2014 software, black edition (Carl Zeiss, Germany). Subsets were created for the medial sections of individual root zones corresponding to a respective Z-stack for the study of ANN1-GFP distribution and measuring fluorescence intensity along the medial plane. ANN1-GFP localization was depicted also in transversal sections of individual root tip zones that were obtained from orthogonal projections of imaged roots. The quantification of the intensity profile was carried out along the medial plane of the corresponding radial zonation but perpendicular to the medial axis. Several subsets were made to embrace whole cells: three trichoblasts, three atrichoblasts, and three root hairs. Subsets were transformed by using maximum intensity projection from individual optical sections. Although quantification of fluorescence intensities is not influenced by post-acquisition intensity adjustments, brightness, and contrast of images were uniformly corrected before the fluorescence intensity measurement and subsequent statistical evaluation. Measurements were carried out along three different planes of individual cells. All data were obtained from three biological repetitions of three independent transgenic plants. Fluorescence intensities were subjected to one-way ANOVA with post-hoc Tukey HSD test at an online web statistical calculator (http://astatsa.com/OneWay_Anova_with_TukeyHSD/). Statistical significance was considered at the level of P value lower than 0.05. Production of plots was carried out with Microsoft Excel software.

### Confocal and Spinning Disk Microscopy

Overview of ANN1-GFP and free GFP distribution in 3- and 7-day-old *Arabidopsis* plants were studied using LSM 710 (Carl Zeiss, Germany) equipped with Plan-Apochromat 20×/0.8 NA (Carl Zeiss, Germany), Plan-Apochromat 40×/1.4 NA Oil DIC (Carl Zeiss, Germany), and alpha Plan-Apochromat 63×/1.46 NA Oil (Carl Zeiss, Germany) objectives. Live imaging of ANN1-GFP in dividing petiole epidermal cells of 4- to 5-day-old *Arabidopsis* plants was carried out with a spinning disk microscope (Cell Observer SD, Carl Zeiss, Germany) equipped with Plan-Apochromat 20×/0.8 NA and 63×/1.4 NA oil immersion objectives (Carl Zeiss, Germany). Samples were imaged with a 488 nm excitation laser line and BP525/50 emission filter for detection of GFP. The scanning was performed every 60 s for a total time of 60 min. Images were recorded with an Evolve 512 EM-CCD camera with the exposure time of 100 ms. Image post-processing and deconvolution using the constrained iterative algorithm with default settings were performed using ZEN 2010 software.

### Life Imaging of ANN1-GFP Localization During Plasmolysis

For *in vivo* localization study of ANN1-GFP during plasmolysis, 3- to 4-day-old *Arabidopsis* plants expressing *proANN1::ANN1:GFP* or *35S::sGFP* (control) constructs were used. Samples were mounted in half-strength MS medium (or in half-strength MS medium with FM4-64) in micro-chambers, and hypocotyl epidermal or primary root cells were documented at control conditions. Osmotic stress was induced either with 500 mM NaCl or 1 M mannitol solutions for hypocotyl, and 250 mM NaCl or 500 mM mannitol for root (in half-strength MS), which were applied by perfusion in a volume of about 100–150 µl. Imaging of ANN1-GFP or free GFP relocations during plasmolysis was done within the range of 1–40 min. Subsequently, deplasmolysis was realized by thorough sample perfusion with half-strength MS. Experiments were conducted by Airyscan CLSM 880 (Carl Zeiss, Germany) equipped with 20×/0.8 NA dry Plan-Apochromat objective (Carl Zeiss, Germany). Samples (hypocotyl in ANN1-GFP line) were imaged with a 488-nm excitation laser line and BP420-480 and BP495-550 emission filters for GFP detection. The image post-processing was done using ZEN 2014 software (Carl Zeiss, Germany). Additionally, Photoshop 6.0/CS, Microsoft Excel, and PowerPoint were used for final figure plates preparation.

### FM4-64 Staining

The membrane styryl dye FM4-64 [N-(3-triethylammoniumpropyl)-4-(6-(4-(diethylamino)phenyl)hexatrienyl) pyridinium dibromide; Thermo Fisher Scientific, Waltham, MA, United States] was used for colocalization study of ANN1-GFP or free GFP (control) with the plasma membrane in root cells. Three-day-old *Arabidopsis* seedlings were placed in a drop of half-strength MS culture medium with 4 μM FM4-64 on a microscope slide in darkness for 15 min. After imaging under control conditions for approximately 15 min, the sample was washed by perfusion with the dye-free half-strength MS medium containing NaCl or mannitol and observed further. Specimens were observed by Airyscan CLSM 880 (Carl Zeiss, Germany) equipped with 20×/0.8 NA dry Plan-Apochromat objective (Carl Zeiss, Germany) and imaged using 488-nm excitation laser line and BP420-480 and BP495-550 emission filters for GFP detection, while FM4-64 was detected using beam splitter MBS 488/561 and BP420-480 and LP605. After the post-processing of images, fluorescence intensities of ANN1-GFP, free GFP, and FM4-64 were measured at the interface along two adjacent epidermal cells in the elongation zone of the primary root in ZEN 2014 software (Carl Zeiss, Germany). Photoshop 6.0/CS, Microsoft Excel, and PowerPoint were used for final figure plate preparation.

### Whole-Mount Immunofluorescence Labeling

Immunolocalization of microtubules and ANN1-GFP in 3-day-old *Arabidopsis* seedling roots was performed according to the published protocol of Šamajová et al. (2014). Samples were immunostained sequentially with the mouse monoclonal anti-GFP primary antibody (Abcam) diluted 1:100 and incubated at 4°C overnight, followed by Alexa-Fluor 488 rabbit anti-mouse as the secondary antibody diluted 1:500 for 1.5 h at 37°C and next 1.5 h at RT. Second immunolabeling was performed with rat anti–α-tubulin antibody (clone YOL1/34, Bio-Rad) diluted 1:300 at 4°C overnight and followed by secondary antibody Alexa-Fluor 647 goat anti-rat diluted 1:500 for 1.5 h at 37°C and 1.5 h at RT. All antibodies were diluted in PBS containing 3% (w/v) BSA. Nuclei were counterstained with DAPI. Microscopic analysis of immunolabeled samples was performed using microscopy platform LSM880 with Airyscan equipped with a 32 GaAsP detector (Carl Zeiss, Germany) and single-photon excitation with the 405 nm for DAPI, 488 nm for Alexa-Fluor 488, and 633 nm for Alexa-Fluor 647 detection. The image post-processing was done using ZEN 2014 and final figure plates were obtained using Photoshop 6.0/CS and Microsoft PowerPoint software.

### Analysis of mRNA Expression Levels by Quantitative Real-Time PCR

RNA Isolation Reagent (TRI Reagent from Sigma-Aldrich) was used to isolate total RNA from wild-type Col-0 and *proANN1::ANN1:GFP* plants (root and aerial part separately) 5 days after germination. The procedure was performed according to the kit manufacturer’s instructions. The purity of RNA was verified using NanoDrop (Thermo Scientific, Waltham, United States). DNA was degraded by DNase I at 37°C for 40 min. After the deactivation of the enzyme with EDTA, the reverse transcription was carried out. In the first step, 0.5 μl of oligo-dt-primers annealed at 70°C for 10 min on the RNAs (500 ng/reaction), subsequently the reaction was inhibited at 70°C for 10 min and cold down on ice. Complementary DNA of the gene *AtANN1* (At1g35720.1) was synthesized in the reaction consisting of 10 μl of RNA mix with annealed oligos and 4 μl of M-MLV Reverse Transcriptase 5x reaction buffer (Promega, Madison WI, USA), 1 μl of dNTP mix (10 mM), 0.4 μl (16 units) RNasin^®^ Plus RNase inhibitor (Promega), 0.4 μl (40 units) M-MLV Reverse Transcriptase (Promega), and H_2_O (PCR grade) in the total volume of 20 μl. PCR reaction was performed at 42°C for 3 h and at 70°C 10 min (for inactivation of reverse transcriptase). Complementary DNA was diluted 50x for quantitative RT-PCRs, which were carried out in 96-well plate with StepOnePlus Real-time PCR system (Applied Biosystems, CA, United States). Double strand DNA synthesis was monitored using SYBR^®^ Green, and the reaction mixture contained 5 μl of Power SYBR^®^ Green PCR master mix (Thermo Fisher Scientific), 10 μM gene-specific primers (**Supplementary Table 2**), and 2.5 μl of cDNA. The standard thermal profile used was 95°C for 10 min, 40 cycles of 95°C for 15 s, and 60°C for 1 min. Experiments were repeated three times. Expression data were normalized to the expression of a reference gene *GAPDH (GLYCERALDEHYDE-3-PHOSPHATE DEHYDROGENASE*; At1g13440). Relative gene expression was calculated using 2^−ΔΔ^ *^Cq^* method.

## Results

### Developmental Localization Pattern of ANN1-GFP in *Arabidopsis* Roots

*AtANN1* together with putative native promoter region were amplified from genomic DNA obtained from *Arabidopsis thaliana* (L.) ecotype Columbia (Col-0). Using restriction cloning, we engineered a fusion construct *ANN1:GFP* expressed under the control of *ANN1* native promoter (*proANN1::ANN1:GFP*). The presence and abundance of the fusion protein ANN1-GFP were verified by western blotting with a monoclonal anti-GFP antibody separately in roots (**Figure S1A**) and aerial plant parts (**Figure S1B**). The presence of a single band at 62 kDa corresponded to the molecular weight of the ANN1-GFP fusion protein (**Figure S1**). In addition, *ANN1* transcript levels were evaluated in roots and aerial plant parts using quantitative RT-PCR analysis, revealing no significant differences between control Col-0 and transgenic lines carrying *proANN1::ANN1:GFP* construct (**Figure S2**).

**Figure 1:**
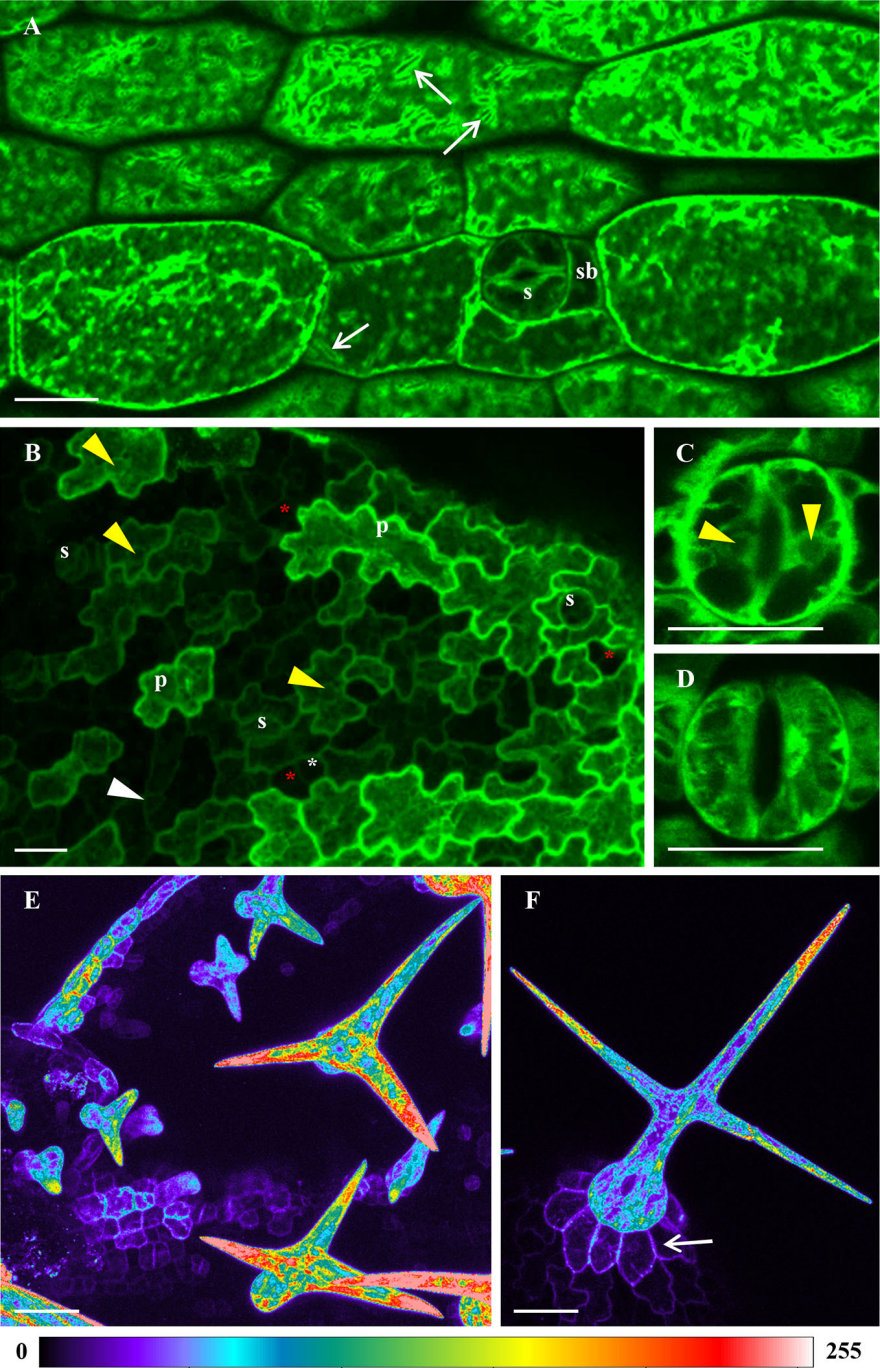
Tissue- and cell-specific localization of ANN1-GFP in different aerial organs of *A. thaliana* seedlings stably expressing *proANN1::ANN1:GFP* construct using confocal laser scanning microscopy (CLSM). **(A)** Expression of ANN1-GFP in hypocotyl epidermal cells showing its localization in the cell cortex, cytoplasmic strands, and in areas around membranous compartments resembling ER bodies (arrows). ANN1-GFP expression delineated cell cortex in stomata guard cells (s) and subsidiary cells (sb). **(B)** Unequal distribution of ANN1-GFP in epidermal cells of developing first true leaf. Prominent production of ANN1-GFP was evident in expanding pavement cells (p), whereas it was almost absent in stomatal precursor cells: meristemoid mother cells (red asterisks), meristemoids (white arrowhead) and guard mother cells (white asterisks). **(C, D)** Distribution of ANN1-GFP within radially organized arrays of cytoplasmic strands in leaf stomata guard cells presented in the medial plane **(C)** and in the cortical plane **(D)**. Note the absence of ANN1-GFP fluorescence in nuclei indicated by yellow arrowheads in **(B, C)**. **(E, F)** Color-coded images of ANN1-GFP localization in different developmental stages of trichomes. Note the tip-focused accumulation of ANN1-GFP in trichomes **(E, F)** and its lower abundance in basal cells of developed trichome indicated by an arrow in **(F)**. Scale bars = 20 μm.

**Figure 2:**
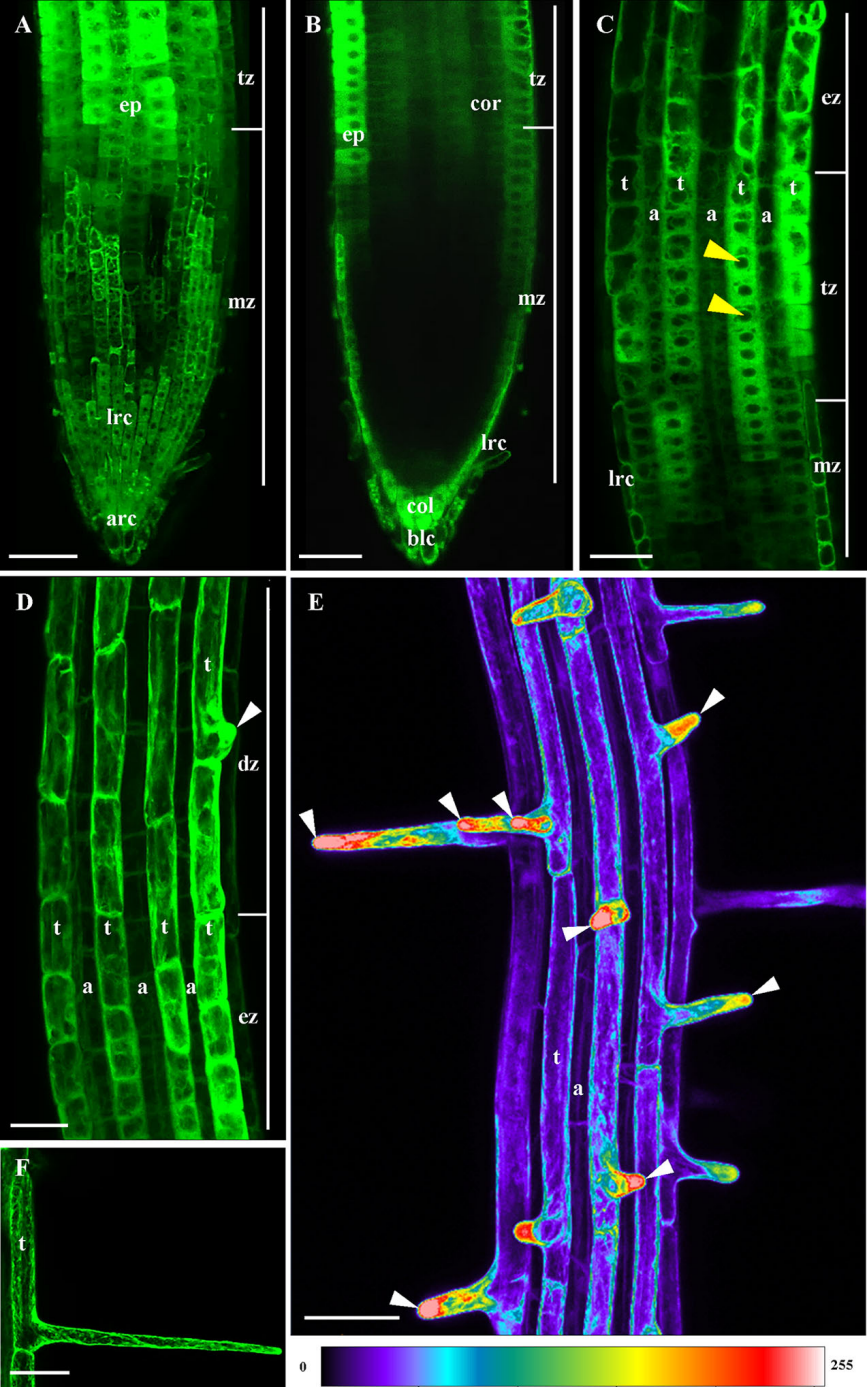
Localization of ANN1-GFP in the primary root of *A. thaliana* stably expressing *proANN1::ANN1:GFP* construct using CLSM. **(A)** Maximum intensity projection and **(B)** medial optical section overview of ANN1-GFP localization in the root apex. In the meristematic zone (mz) the expression level is very low. ANN1-GFP starts to be detectable in root epidermis (ep) within the distal portion of the meristematic zone (mz) and in the transition zone (tz), where ANN1-GFP signal appears also in deeper root tissues. **(A)** Relatively high expression level of ANN1-GFP is present in apical root cap cells (arc) and lateral root cap cells (lrc). **(B)** Apparent accumulation of ANN1-GFP is also in border-like cells (blc) and the apical layer of columella (col) in medial plane of the root. **(C, D)** A striped-like pattern of ANN1-GFP expression in root epidermis within the elongation **(C)** and differentiation zone **(D)** of the root. A pattern of higher expression in trichoblasts (t) and lower expression in atrichoblasts (a) is preserved from the end of meristematic zone (mz) through the transition zone (tz) to the elongation zone (ez). The abundance of ANN1-GFP in trichoblast cell files is enhanced before and during the root hair initiation within the differentiation zone (dz), when ANN1-GFP is accumulated in the bulge and emerging root hair tip [arrowhead in **(D)**]. Note the absence of ANN1-GFP fluorescence in nuclei of trichoblasts indicated by yellow arrowheads in **(C)**. **(E)** A color-coded image representing maximum intensity projection from individual Z-stacks of root differentiation zone with moderate fluorescence signal in trichoblast cell files (t), high fluorescence signal in emerging and growing root hairs, whereas low signal was observed in atrichoblast cell files (a). Note the tip-focused accumulation of ANN1-GFP in emerging and actively-growing root hairs indicated by arrowheads. **(F)** Typical net-like distribution of ANN1-GFP in cortical layers of cytoplasm in the trichoblast (t) and fully-grown root hair. Scale bars = 50 μm.

After confirmation of ANN1-GFP expression, we studied in detail its developmental tissue-specific and subcellular localization patterns in *Arabidopsis* plants. CLSM provided a useful tool with sufficient spatial resolution to monitor the overall distribution of ANN1-GFP in the whole plant body (**Figures 1** and **2**). Using this method, we acquired cell-specific expression and subcellular localization of ANN1-GFP in aerial parts of *Arabidopsis* seedlings (**Figure 1**). In hypocotyl epidermal cells, ANN1-GFP was localized in the cortical cytoplasm and more dense fluorescence delineated membranous compartments resembling endoplasmic reticulum (ER) bodies, suggesting a possible role in endomembrane trafficking (**Figure 1A**). ANN1-GFP was observed in the cortical cytoplasm close to the PM and in cytoplasmic strands of pavement and stomatal guard cells of leaf epidermis, but it was excluded from nuclei in these cell types (**Figures 1B–D**). Some stomatal lineage cells including meristemoid mother cells, meristemoids, and guard mother cells seemed to lack the ANN1-GFP signal (**Figure 1B**). Nevertheless, in stomatal guard cells, ANN1-GFP was localized around nuclei, in the cortical cytoplasm, and in radially organized cytoplasmic strands (**Figures 1C, D**). ANN1-GFP was also enriched in leaf trichomes showing net-like distribution (**Figures 1E, F**), that resembled an arrangement of ER. The expression level of the fusion protein was much lower in trichome basal cells (**Figure 1F**). Detailed examination of diverse trichome developmental stages using color-coded images revealed that ANN1-GFP accumulated preferably in the tips of growing trichome branches (**Figures 1E, F**).

Imaging on *Arabidopsis* roots showed that ANN1-GFP was abundant in particular root cell types. Maximum intensity projection from individual Z-stacks of the primary root revealed very strong ANN1-GFP expression level in the apical and lateral part of the root cap and in the epidermis within the root transition zone (**Figures 2A, B**). However, the signal was almost missing in the root cortex, the endodermis, and in the central cylinder of the root meristematic zone. A weak signal of ANN1-GFP started to be detectable in the inner tissues of the root transition zone (**Figure 2B**). In root epidermis, the markedly stronger ANN1-GFP expression level in trichoblasts as compared to atrichoblasts was present in the distal part of the root meristematic zone and in transition and elongation root zones (**Figures 2C, D**). ANN1-GFP strongly accumulated in the bulge during root hair initiation (**Figures 2D, E**) and in tips of growing root hairs (**Figures 2E, F**) as documented by color-coded overview image on root hair formation zone (**Figure 2E**), suggesting a potential role in root hair tip growth.

Imaging of free GFP in *Arabidopsis* under identical conditions by CLSM was used as a control. Unlike ANN1-GFP, which was depleted from nuclei, free GFP accumulated in nuclei of all examined cell types (**Figure S3**).

In order to study ANN1-GFP developmental localization in *Arabidopsis* roots growing at natural orientation (in accordance with gravity vector), we have used advanced LSFM (Ovečka et al., 2015; Ovečka et al., 2018). Importantly, this method allowed developmental cell- and tissue-specific imaging of ANN1-GFP distribution in growing roots (including deep root tissues) with high spatial and superb temporal resolutions over prolonged period of time. Experimental plants expressing *proANN1::ANN1:GFP* construct were growing continuously under controlled physiological conditions directly in the microscopic chamber. We imaged three living plants individually in 5 min time-point intervals for 7–15 h. Acquisition of three related image views co-aligned along the y-axis were arranged in order to capture complete view on growing root tips over long period of time (**Videos S1–S3**).

**Figure 3:**
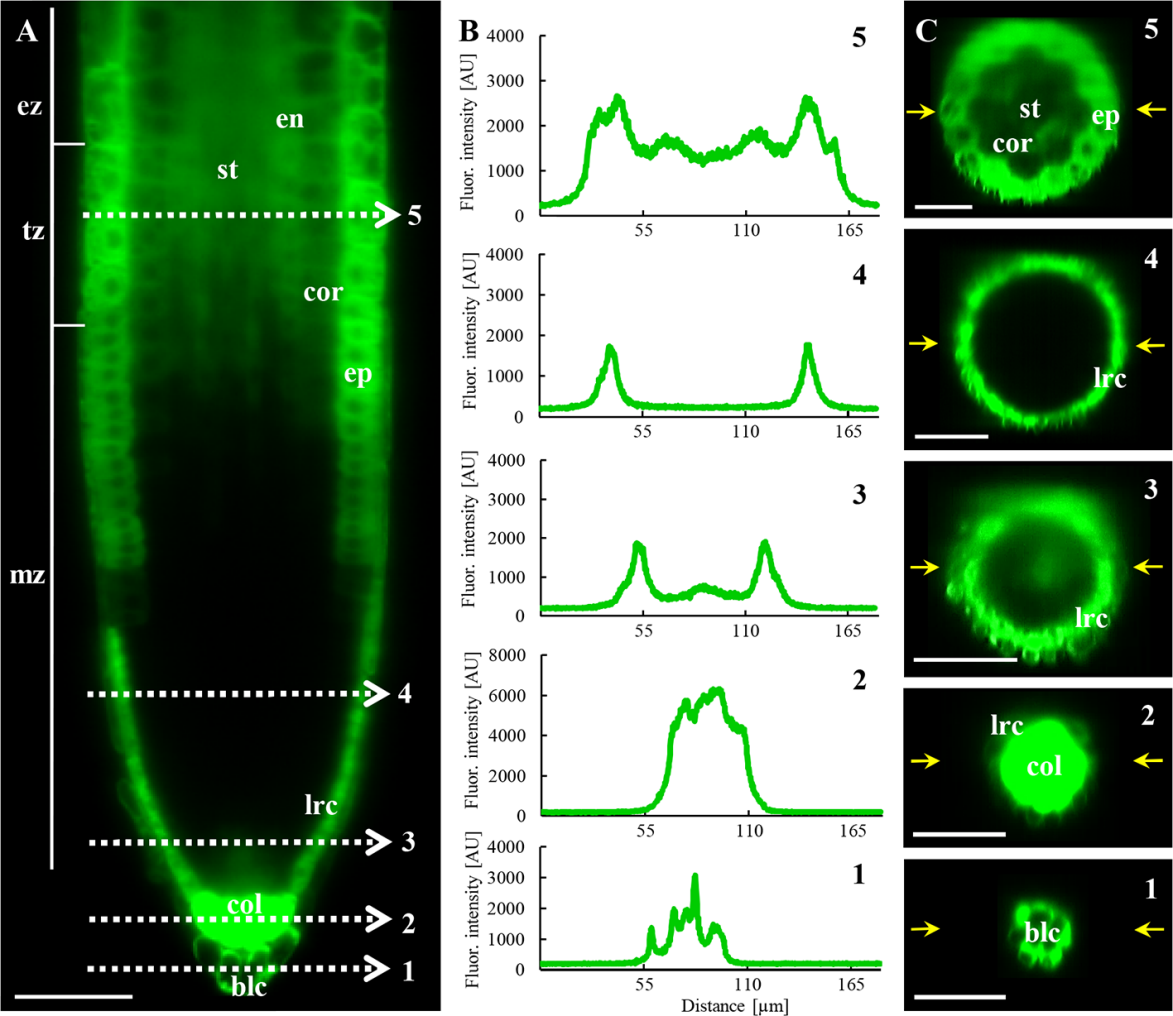
*In vivo* localization of ANN1-GFP in meristematic and transition zone of primary root in *A. thaliana* expressing *proANN1::ANN1:GFP* construct using LSFM. **(A)** Medial optical section of the primary root showing the distribution pattern of ANN1-GFP expression. Strong level of ANN1-GFP production is visible in the columella cells (col) and in lateral root cap cells (lrc). Cells of the root meristematic zone (mz) are devoited of ANN1-GFP. The signal of ANN1-GFP is enhanced in epidermal cells (ep) within the meristematic zone and stays strong through the transition (tz) to elongation zone (ez). Starting in the transition zone (tz) ANN1-GFP is produced also in inner root tissues: cortex (cor), endodermis (en), and stele (st). White dashed lines (1–5) indicate the position of the profiles along which the fluorescence intensities, showed in **(B)**, were measured for quantitative evaluation of ANN1-GFP distribution. **(C)** Distribution of ANN1-GFP visualized in radial root sections prepared from orthogonal projections of the root apex at the position of respective fluorescence intensity profiles in **(A)**, indicated by yellow arrows. Scale bars = 50 µm.

**Figure 4:**
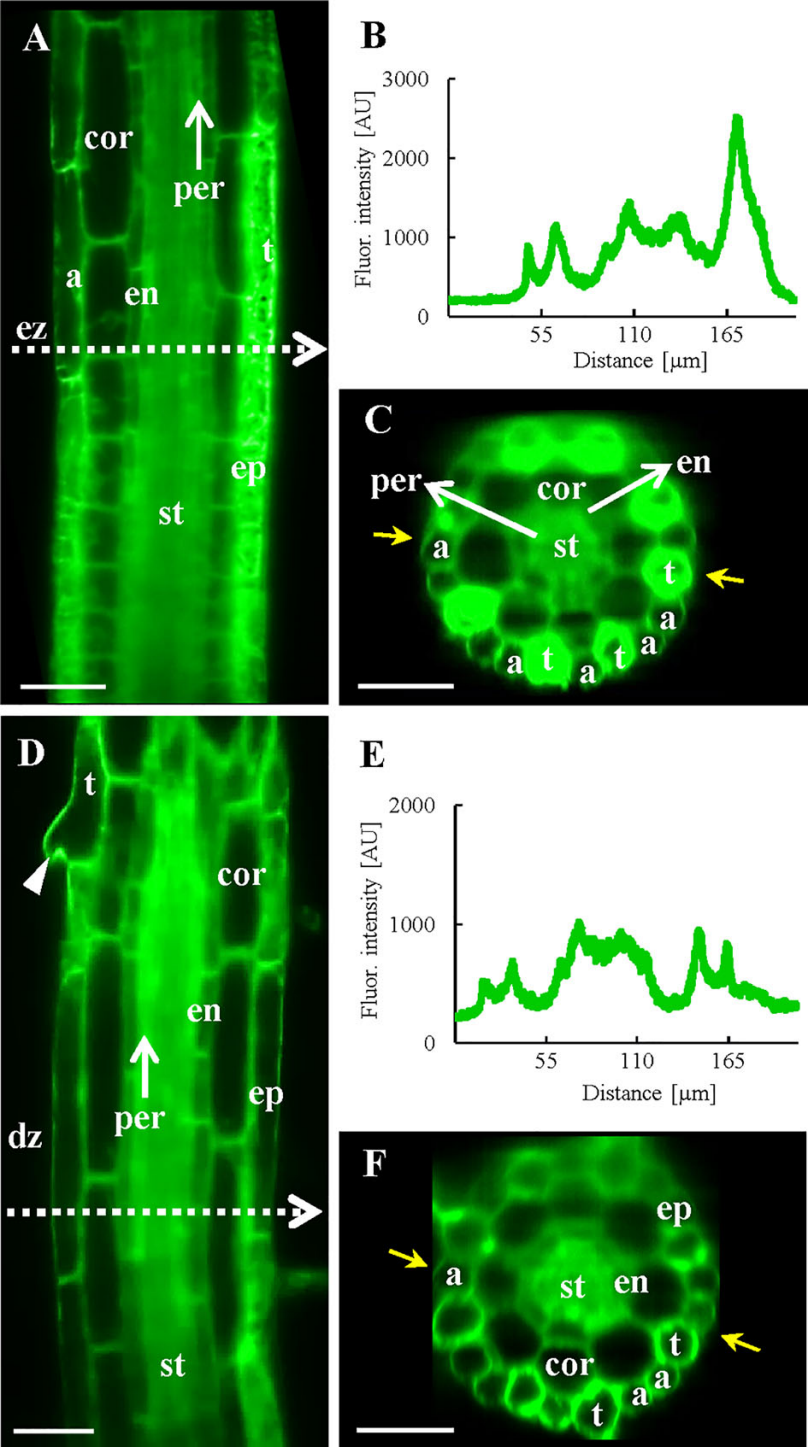
*In vivo* localization of ANN1-GFP in elongation and differentiation zone of the primary root in *A. thaliana* expressing *proANN1::ANN1:GFP* construct using LSFM. **(A)** Medial optical section of the elongation zone shows tissue-specific localization pattern of ANN1-GFP, with higher amount in trichoblasts (t) in comparison to atrichoblasts (a) of the epidermis (ep), lowering of the ANN1-GFP content in vacuolated cortical cells (cor) and in endodermal cells (en), and rather homogenous distribution in the stele (st) and pericycle (per) cells. White dashed arrow indicates the position where the fluorescence intensity profile has been measured. **(B)** The fluorescence intensity along the profile shown in **(A)** and yellow arrows in **(C)**. **(C)** The corresponding orthogonal projection of the radial root section at the position of the intensity profile in **(A)**. **(D)** Medial optical section of the differentiation zone with the strongest level of ANN1-GFP production in the stele (st) and decreased amount in vacuolated cells of endodermis (en), cortex (cor), and epidermis (ep). Trichoblast with emerged root hair is marked by an arrowhead. White dashed arrow indicates the position where the fluorescence intensity profile has been measured. **(E)** The fluorescence intensity along the profile shown in **(D)** and yellow arrows in **(F)**. **(F)** The corresponding orthogonal projection of the radial root section at the position of the intensity profile in **(D)**. Scale bars = 50 µm.

Besides the fluorescence visualization in the root epidermis, LSFM provided a very good tool for deep imaging and localization of ANN1-GFP also in inner root tissues (**Figures 3** and **4**). Consistently with data from CLSM, longitudinal optical sections of the root apex from LSFM showed the highest fluorescence intensity of ANN1-GFP in root cap and root epidermis (**Figure 3A**). The strongest signal in root cap was present in lower cell row of columella, columella-related border-like cells, and lateral root cap cells (**Figure 3A**). ANN1-GFP was almost missing in the meristematic root zone with exception of epidermis (**Figure 3A**). Semi-quantitative analysis of tissue-specific ANN1-GFP fluorescence intensity distribution in different zones of primary root confirmed high level of ANN1-GFP amount in columella-related border-like cells (**Figures 3A–C**; profile 1). Noticeably highest level of homogenous ANN1-GFP production was evident in lower cell row of the columella (**Figures 3A–C**; profile 2). However, fluorescence signal intensity was almost three-times lower in central columella cells, which was even not reaching the fluorescence intensity present in lateral root cap cells (**Figures 3A–C**; profile 3). In comparison to columella, only weak and non-uniform signal was observed at the area of quiescent center and stem cells niche. Virtually, no fluorescence was detected throughout the meristematic zone, where the fluorescence detected originated only from lateral root cap cells (**Figures 3A–C**; profile 4). Prominent ANN1-GFP expression in the epidermis started within the distal portion of the meristematic zone and in the transition zone, accompanied by weak appearance of ANN1-GFP signal in all inner tissues including cortex, endodermis, pericycle, and stele in the transition zone (**Figures 3A–C**; profile 5). On the contrary, more equal distribution of fluorescence intensities in different cell layers of the elongation (**Figures 4A–C**) and differentiation zone (**Figures 4D–F**) were obvious. To reveal a general expression pattern of ANN1-GFP during primary root development, a quantitative evaluation of ANN1-GFP fluorescence distribution was performed in regarding of individual root zones. Fluorescence intensity measurements along the profile of medial optical plane of the root apex (**Figure S4A**) and perpendicularly to the longitudinal root axis corresponding to root cross-sections (**Figure S4B**) revealed low level of fluorescence intensity in the meristematic zone and significantly increased values in the transition, elongation, and differentiation zones of the root (**Figure S4**).

Fluctuations of the fluorescence intensity using the profile measurements in transition, elongation and differentiation root zones corresponded to substantially higher expression level of ANN1-GFP in trichoblasts than in atrichoblasts (**Figures 3** and **4**). Next, we focused on spatio-temporal localization of ANN1-GFP during the root hair development (**Figure S5**). The root hair initiation was proceeded by dynamic relocalization of homogenously distributed ANN1-GFP in the cytoplasm to the basal part of the trichoblast where the bulge will be formed (**Figure S5**; **Video S4**). Interestingly, ANN1-GFP was strongly accumulated in growing root hair, which was accompanied by decreased ANN1-GFP abundance in trichoblast cell (**Figures 2E** and **S5**). Fluorescence signal in trichoblasts before root hair initiation was significantly higher than after root hair outgrowth. On the other hand, the lowest fluorescence signal was observed in atrichoblasts (**Figure S5D**). High expression, dynamic relocation, and specific accumulation pattern of ANN1-GFP in trichoblast cell files clearly indicate active involvement of ANN1 in the process of root hair formation.

**Figure 5:**
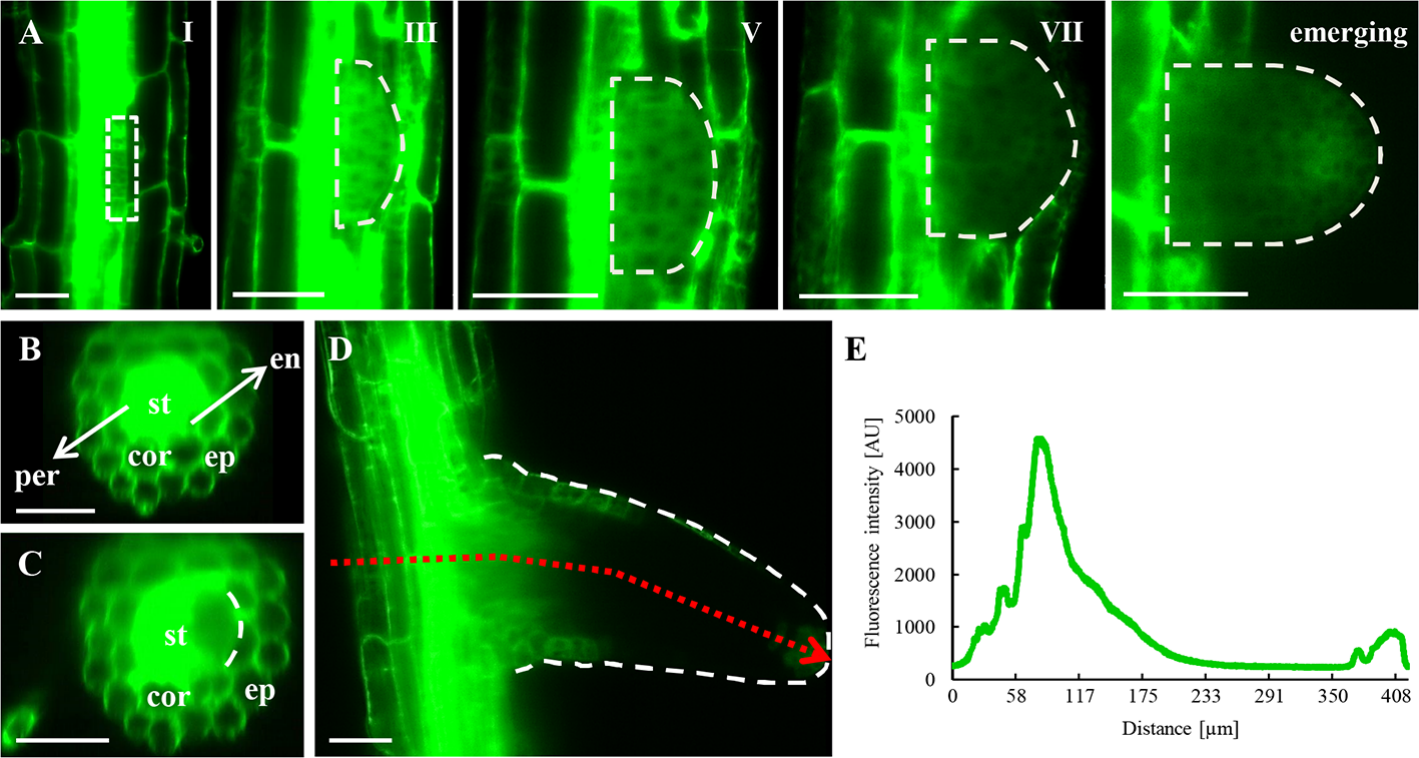
*In vivo* localization of ANN1-GFP during lateral root formation and emergence in transgenic *A. thaliana* expressing *proANN1::ANN1:GFP* construct using LSFM. **(A)** Representative images of selected developmental stages of lateral root primordium (LRP) establishment. ANN1-GFP signal was localized in the cytoplasm and was absent in nuclei of LRP cells in early stages of LRP formation (phases I, III, and V). ANN1-GFP signal considerably decreased in LRP cells in the phase VII and during lateral root emergence. **(B)** Cross-section of the primary root from orthogonal projection before LRP initiation with the strong and uniformly distributed signal of ANN1-GFP in the stele (st) and decreased amount in vacuolated cells of endodermis (en), cortex (cor), and epidermis (ep). **(C)** Drop of the ANN1-GFP signal intensity in the pericycle and adjacent stele tissue at the time of LRP establishment. **(D)** Distribution of ANN1-GFP signal in the emerging lateral root. Tissue-specific fluorescence intensity in emerging lateral root was determined along the profile representing a cross-section of the primary root and longitudinal gradient in developing lateral root (as shown by dotted red arrow). **(E)** The fluorescence intensity along the profile shown in **(D)**. Scale bars = 50 μm.

Long-term imaging of growing roots in LSFM allowed the observation of ANN1-GFP distribution during lateral root development (**Figure 5**; **Video S5**). During the early phases of the lateral root primordia (LRP) formation (**Figure 5A**, stages I, III, and V), the strong ANN1-GFP signal was homogenously distributed in the cytoplasm of LRP cells. However, gradual decrease in the cellular ANN1-GFP expression level was observed in later LRP developmental phases (**Figure 5A**, stage VII) and in cells of emerging lateral roots (**Figure 5A**, stage emerging). Strong and equal signal of ANN1-GFP was present in endodermis, pericycle, and stele before LRP initiation, as shown in orthogonal projection of the respective part of the primary root (**Figure 5B**). Interestingly, with appearance of LRP founder cells ANN1-GFP signal dropped in this area (**Figure 5C**). Tissue-specific and developmental distribution of ANN1-GFP in emerging lateral root resembled the pattern observed in the primary roots (**Figure 5D**). The fluorescence intensity measurement confirmed the absence of ANN1-GFP in the lateral root meristem and its presence in root epidermal and columella cells (**Figure 5E**).

**Figure 6:**
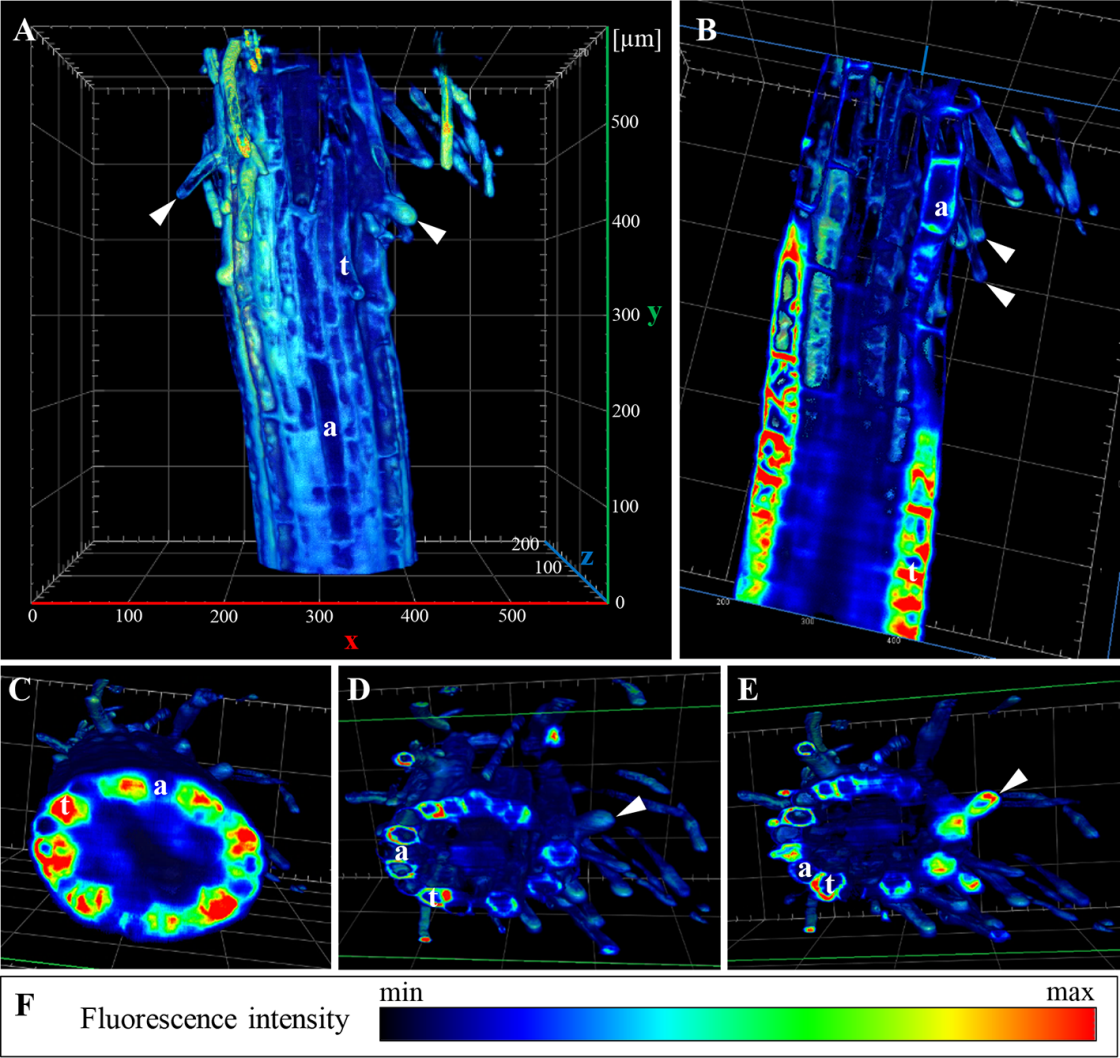
3-D rendering of ANN1-GFP distribution in *A. thaliana* primary root stably expressing *proANN1::ANN1:GFP* construct. Data were obtained by Luxendo MuVI SPIM and after post-processing 3-D reconstruction was made by Arivis. **(A)** Overview of the elongation and differentiation zone of the primary root with root hairs (arrowheads) in different stages of their development. **(B)** Clipping of the 3-D model against z-plane revealed the highest fluorescence intensity of ANN1-GFP (red in pseudocolored intensity scale) in trichoblast cell files (t). **(C–E)** Clipping of the 3-D model against y-plane, where in **(C)** the lowest plane shows root cross-section at the elongation zone with a different distribution of ANN1-GFP in trichoblasts (t) and atrichoblasts (a). **(D, E)** 3-D projections displaying root cross-sections at the differentiation zone, arrowhead indicates growing root hair, whereas y-clipping along its longitudinal plane point to ANN1-GFP accumulation in root hair tip. **(F)** Heat map presents ANN1-GFP fluorescence intensity in pseudocolors with the lowest fluorescence intensity corresponding to black and highest fluorescence intensity corresponding to red color. Arrowheads point root hair tips, (t) points trichoblasts, and (a) atrichoblasts.

Spatial distribution of ANN1-GFP in all developmental zones of young *A. thaliana* primary roots was studied using commercial microscope Luxendo MuVi SPIM allowing to acquire four different views for subsequent multiview fusion. Post-processed data were used as an input for 3-D reconstruction in Arivis software (**Figure 6**). The reconstruction visualized not only surface but also deep tissues of the primary root within the root elongation and differentiation zone, providing a comprehensive overview of the spatial ANN1-GFP distribution (**Video S6**).

**Figure 7:**
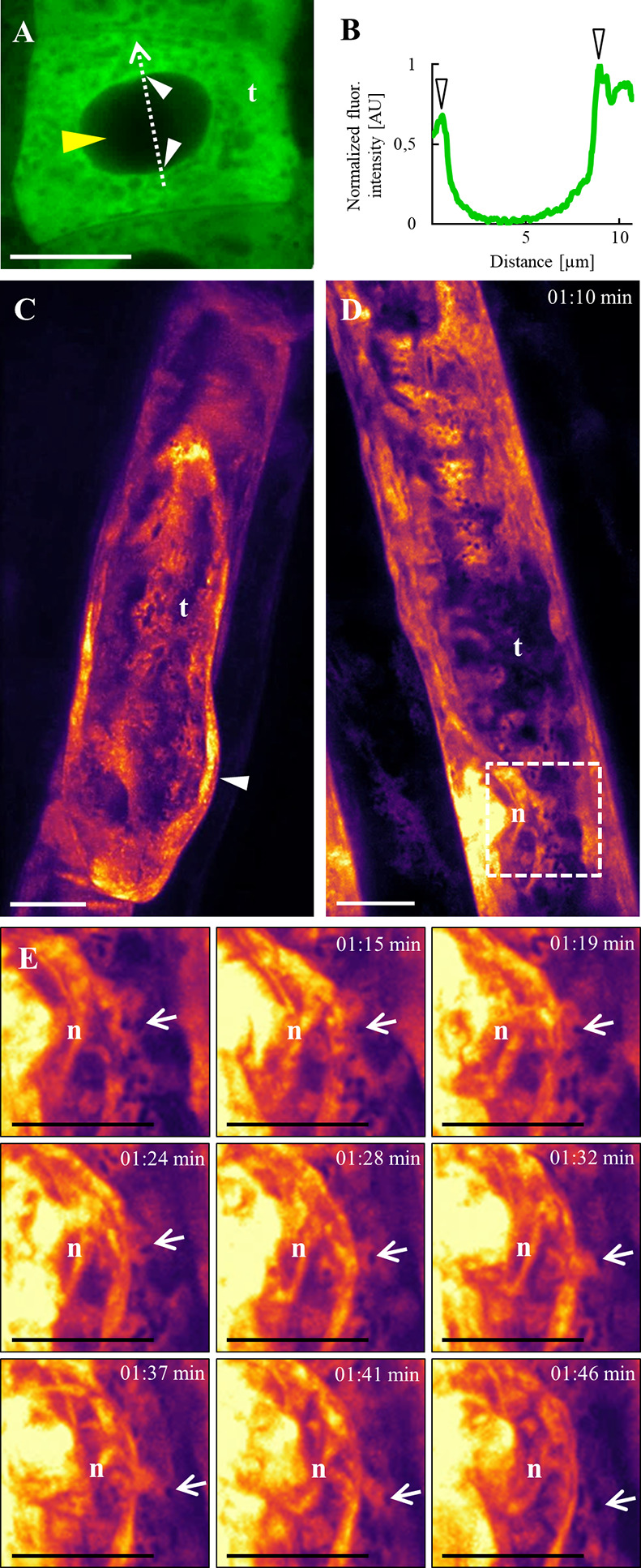
Subcellular localization of ANN1-GFP in trichoblasts using Airyscan CLSM **(A, B)** and lattice LSFM **(C–E)**. **(A)** Accumulation of ANN1-GFP around the nucleus, close to the nuclear envelope (white arrowheads) in trichoblast (t) cell within meristematic root zone. Note the absence of ANN1-GFP fluorescence in the nucleus indicated by the yellow arrowhead. **(B)** Fluorescence intensity profile of ANN1-GFP distribution was measured along a dotted arrow in **(A)** and normalized. Note that arrowheads indicate maximum of measured fluorescence intensity corresponding to ANN1-GFP accumulation around the nuclear envelope. **(C–E)** *In vivo* time-lapse imaging of ANN1-GFP localization in trichoblasts using lattice LSFM. **(C)** Higher fluorescence intensity of ANN1-GFP at the basal end of trichoblast (t) and its accumulation in the cortical cytoplasm of the established bulge (white arrowhead). **(D)** Accumulation of ANN1-GFP around the nucleus (n), close to the nuclear envelope in elongating trichoblast (t). **(E)** Magnified boxed area in **(D)** showing selected stills of vesicular dynamic movement (arrows) around the nucleus (n) of elongating trichoblast. Scale bars = 10 μm.

Using higher resolution microscopy techniques such as Airyscan CLSM and lattice LSFM, we observed a more detailed subcellular localization pattern of ANN1-GFP in root trichoblast cells (**Figure 7**; **Videos S7–S8**). ANN1-GFP accumulated around the nucleus, close to the nuclear envelope in trichoblast cells within meristematic and elongation root zones (**Figures 7A, B, D, E**). Moreover, in the course of nuclear migration within the cell, the increased fluorescence intensity of ANN1-GFP streamed close to the nuclear envelope. A semi-quantitative analysis showed the signal peak on the nuclear envelope, which is continuous with ER (**Figures 7A, B**). This documented dynamic properties of ANN1-GFP subcellular relocation together with nucleus migrating toward the basal pole of the cell before bulge formation (**Figures 7C, D**; **Video S7**). After the bulge has been created, the signal of ANN1-GFP was enriched at the cortical area of the bulge (**Figure 7C**; **Video S8**).

**Figure 8:**
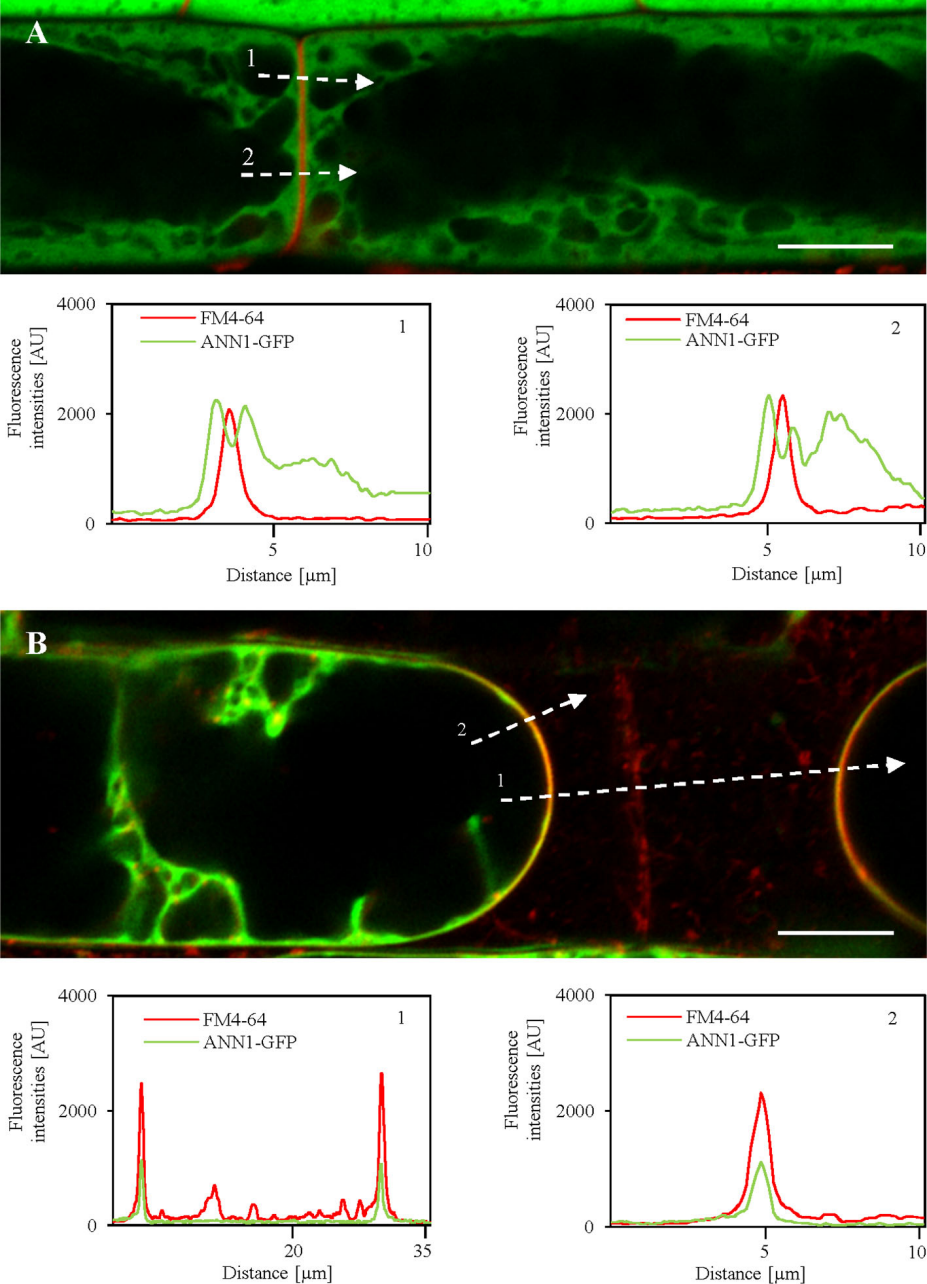
Relocation of ANN1-GFP in plasmolyzed root epidermal cells to the FM4-64-labeled plasma membranes after salt stress as revealed by high-resolution Airyscan CLSM. **(A)** Root epidermal cells in the elongation root zone carrying ANN1-GFP and co-labeled with FM4-64 (red) for detection of the plasma membrane under control conditions. Fluorescence intensity profiles measured along the dotted white lines 1 and 2 in **(A)** showing distinctly separated ANN1-GFP and FM4-64 peaks, suggesting no obvious colocalization. **(B)** Salt stress causes relocation of ANN1-GFP to FM4-64 labeled plasma membranes in plasmolyzed root epidermal cells of elongation root zone. Fluorescence intensity profiles measured along the dotted white lines 1 and 2 in **(B)** showing a strong overlap of FM4-64 and ANN1-GFP peaks, suggesting their colocalization. Scale bars = 10 μm.

Annexins can associate with membrane phospholipids in a calcium-dependent manner and are thought to participate in endomembrane ﬂow (Konopka-Postupolska and Clark, 2017). In order to investigate precise colocalization of ANN1-GFP fusion protein with the plasma membrane (PM), we used lipophilic styryl dye FM4-64 (**Figure 8**). The observation was performed on the primary roots of *A. thaliana* seedlings stably expressing ANN1-GFP using high-resolution Airyscan CLSM. In the root epidermal cells colocalization of ANN1-GFP with FM4-64 labeled PM was confirmed after plasmolysis caused by NaCl but not under control conditions (**Figure 8**). Semi-quantitative evaluation of the fluorescence intensity in plasmolyzed cells showed overlapping peaks of ANN1-GFP fluorescence signals with FM4-64 signal at the PM of retracted protoplasts (**Figure 8B**). Detailed analysis of the fluorescence intensity profiles spanning the plasmolyzed adjacent cells confirmed overlapping fluorescence signals and thus colocalization of ANN1-GFP with FM4-64 at the PM (**Figure 8B**). As a control, we have used a transgenic line carrying free GFP where no colocalization of this free GFP with PM was observed in plasmolyzed cells under identical experimental conditions (**Figure S6**).

Further, osmotic stress-induced relocation of ANN1-GFP was studied in hypocotyl cells treated either with mannitol (**Figures S7A–F**) or NaCl (**Figures S7G–L**) using Airyscan CLSM. Both non-ionic (mannitol) and ionic (NaCl) osmotics caused plasmolysis of hypocotyl cells accompanied by ANN1-GFP relocation and accumulation to the areas of PM retracting from the cell wall, as well as to the Hechtian strands and reticulum. After deplasmolysis, Hechtian strands and reticulum largely disappeared, and retracted protoplasts almost fully recovered, but ANN1-GFP was still enriched at the PM (**Figures S7D, J**). Such osmotic stress-dependent enrichment of ANN1-GFP at the PM and association with Hechtian strands and reticulum indicate its possible osmoprotective role.

### Association of ANN1-GFP With Microtubules During Cell Division

**Figure 9:**
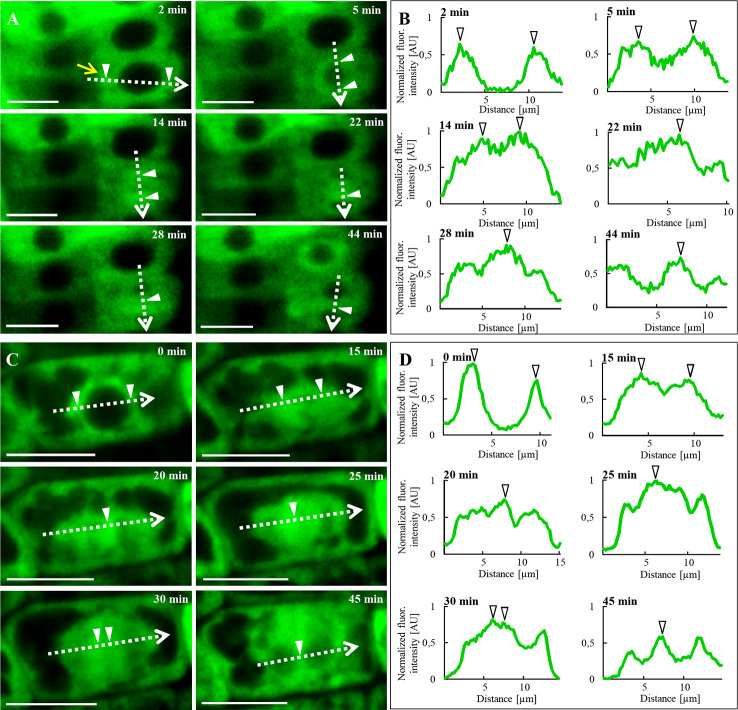
*In vivo* time-lapse imaging of subcellular localization of ANN1-GFP during cell division in transgenic *A. thaliana* expressing *proANN1::ANN1:GFP* construct using LSFM. **(A)** Redistribution of ANN1-GFP in dividing root epidermal cell. At the initial stage (2 min), ANN1-GFP was present at the pre-prophase band of microtubules (yellow arrow). Later on (5 min), ANN1-GFP was enriched around the nucleus before and at the time of mitotic spindle formation (14 min). Next, it relocated to early (22 and 28 min) and late phragmoplast (44 min). **(C)** The analogous pattern of ANN1-GFP relocation and association with mitotic and cytokinetic microtubule arrays was recorded in dividing lateral root cap cell. **(B, D)** Fluorescence intensity profiles of ANN1-GFP distribution were measured along indicated dotted arrows at respective cell division stages. Arrowheads [white in **(A, C)** and black-bordered in **(B, D)**] indicate a maximum of measured fluorescence intensities. Scale bars = 10 μm.

Long-term LSFM imaging allowed to study subcellular localization of ANN1-GFP in cells of root epidermis (**Figures 9A, B**) and lateral root cap cells (**Figures 9C, D**) during cell division (**Video S9**). At the beginning of cell division, ANN1-GFP accumulated in the belt-like cortical structure corresponding to the pre-prophase band of microtubules defining the future cell division plane [**Figures 9A** (2 min), **9C** (0  min)]. Subsequently, ANN1-GFP fluorescence was associated with the mitotic spindle [**Figures 9A** (14 min), **9C** (15 min)]. Later, ANN1-GFP fluorescent signal decorated early phragmoplasts [**Figures 9A** (22 and 28 min), **9C** (25 and 30 min)] and late phragmoplasts, while the midzone occupied by forming cell plate was lacking the GFP signal [**Figures 9A** (44 min), **9C** (45 min)]. When expanding phragmoplast reached the cell periphery, ANN1-GFP relocated to the cytoplasm in daughter cells [**Figures 9A** (44 min), **9C** (45 min)].

Semi-quantitative evaluation of fluorescence intensity measurements along the indicated profiles (**Figures 9A, C**) confirmed the increased association of ANN1-GFP with mitotic and cytokinetic microtubule arrays during individual stages of cell division (**Figures 9B, D**). Analogously, *in vivo* time-lapse imaging using spinning disk microscopy and semi-quantitative evaluation of fluorescence intensity confirmed the close association of ANN1-GFP with mitotic and cytokinetic microtubule arrays in dividing leaf petiole epidermal cells (**Figure S8**; **Video S10**). These results indicate the involvement of annexin 1 in the regulation of cell division.

**Figure 10:**
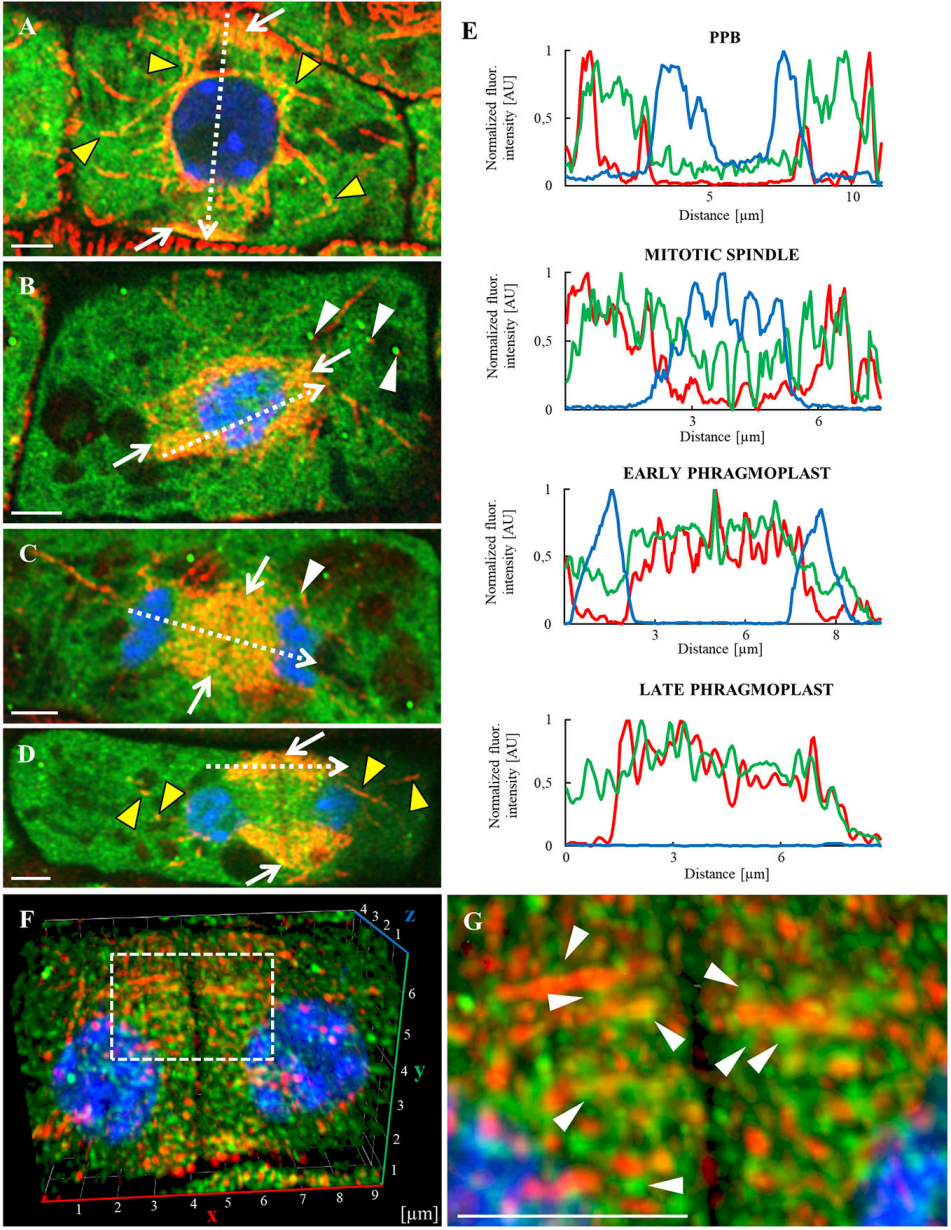
Whole-mount immunoﬂuorescence colocalization study of microtubules (red) and ANN1-GFP (green) in dividing lateral root cap cells made by Airyscan CLSM. Nuclei are labeled with DAPI (blue), while individual division stages (indicated by white arrows) are depicted as merged images: **(A)** Preprophase band (PPB), **(B)** mitotic spindle, **(C)** early phragmoplast, and **(D)** late phragmoplast. Yellow arrowheads indicate the association of ANN1-GFP with microtubules, white arrowheads point to the association of spots containing ANN1-GFP with microtubules. Individual images of microtubules in red, ANN1-GFP in green, and DNA in blue corresponding to merged images **(A–D)** are shown in Figure S10. **(E)** Fluorescence intensity profiles of ANN1-GFP distribution were measured in indicated cell division stages along dotted arrows depicted in **(A–D)** and normalized. Green lines represent ANN1-GFP, red microtubules, and blue DAPI. **(F)** Frontal view of lateral root cap cell (3-D rendered) showing late phragmoplast. **(G)** Detail of ANN1-GFP spot-like localization pattern (white arrowheads) closely associated with phragmoplast microtubules of dotted boxed area in **(F)**. Scale bars = 2 µm.

In order to confirm the role of annexin 1 in cell division and its possible association with microtubules in dividing cells, we performed immunolocalization of ANN1-GFP and microtubules in the primary root tip of *A. thaliana* transgenic line stably expressing *proANN1::ANN1:GFP* construct (**Figures 10** and **S9** and **S10**). Overview of the meristematic zone in the primary root tip revealed dividing cells of the lateral root cap (**Figure S9A**). Orange-colored areas of merged channels indicate the colocalization of green ANN1-GFP signal and red-labeled microtubules. It is evident in the pre-prophase band, mitotic spindle, and phragmoplast (**Figure S9B**). At the pre-prophase stage, as the pre-prophase band progressed in narrowing, individual microtubules were concentrated more closely and were less distinguishable, creating a dense pre-prophase band (**Figures 10A** and **S10A**). During those phases, ANN1-GFP signal was generally cytoplasmic (**Figure S10A**), but some regions, light orange colored in merged channels, indicated colocalization with microtubules (**Figure 10A**). With the creation of mitotic spindle, ANN1-GFP colocalized with microtubules noticeably (**Figure 10B**). Interestingly, we observed colocalization of ANN1-GFP with microtubules also in spot like-manner at random positions (**Figures 10B** and **S10B**). In later stages, ANN1-GFP colocalized with microtubules of early (**Figures 10C** and **S10C**) and late phragmoplast more distinctly (**Figures 10D** and **S10D**). Semi-quantitative evaluation of fluorescence intensity confirmed the overlay of red microtubule signal with green ANN1-GFP signal in the place of the pre-prophase band, in mitotic spindles and in phragmoplasts (**Figure 10E**). Qualitative and quantitative data thus clearly confirmed the colocalization pattern of ANN1-GFP with microtubules during the cell division.

The colocalization pattern between ANN1-GFP and microtubules during cell division was studied in detail. Individual planes acquired from a complete immunostained lateral root cap cell at the late phragmoplast stage were used for 3-D modeling. For the improvement of fluorescence resolution, data were deconvolved using the constrained iterative algorithm with default settings (**Figures 10F, G**; **Video S11**). The frontal view on the 3-D model of dividing lateral root cap cell revealed a characteristic pattern of the phragmoplast organization (**Figure 10F**). A detailed view of the 3-D model showed a close association of phragmoplast microtubules with ANN1-GFP in spot-like localization pattern (**Figure 10G**).

## Discussion

*ANNEXIN* genes encode calcium-dependent phospholipid-binding proteins and represent a multigene family in both plants and animals (Mortimer et al., 2008; Divya et al., 2010). They can function as Ca^2+^-binding and regulating channels or participate in diverse cellular processes including responses to environmental stresses and signaling during plant growth and development (Jami et al., 2012; Yadav et al., 2018; He et al., 2019; Saad et al., 2020).

Up to now, there are only a few works addressing possible ANN1 functions *in vivo* (Konopka-Postupolska et al., 2009; Huh et al., 2010). However, none of them is focused on live imaging of ANN1 subcellular localization and tissue-specific expression in growing and developing plants. *In situ* hybridization, immunolabeling, and autoradiography techniques allowed observation of regulated expression and specific localization pattern of ANN1 in fixed plant samples (Clark et al., 2001; Clark et al., 2005b). In early developmental stages of *Arabidopsis* seedlings, expression and localization of ANN1 were confirmed in epidermal cells of root elongation zone except for root tip, where expression was limited only to the root cap (Clark et al., 2001). In later developmental stages expression of *ANN1* was observed in the whole root (except root meristem) as well in cells of hypocotyl, cotyledon, and apical meristem (Clark et al., 2001). A general overview of developmental expression patterns of ANN1, as well as tissue-specific and subcellular localization presented for the first time in this study, can provide comprehensive guidance for further research focused on this protein. We have used several complementary microscopy platforms including confocal laser scanning (CLSM), spinning-disk (SD), Airyscan (AS), and light-sheet fluorescence microscopy (LSFM) using Z.1, lattice LSFM and Luxendo MuVi SPIM platforms for localization of ANN1-GFP during *Arabidopsis* development. In contrast to CLSM, SD, and AS which may introduce high excitation light intensities causing stress and often also growth inhibition, LSFM overcomes these disadvantages by illuminating the sample by just a thin sheet of light. Aerial parts of seedlings were situated upright in Z.1 LSFM growing chamber with the full freedom to develop continuously in the open aerated tube. Similarly, the root was allowed to grow continuously in solid culture medium used for imaging. Nowadays, only LSFM offers advantages such as the high penetration depth and the possibility of long-term 3-D imaging of a plant sample in near-physiological conditions (Ovečka et al., 2015).

Using all these imaging techniques, we observed very complex ANN1 localization patterns in living and fixed *Arabidopsis* seedlings. For example, ANN1 seems to be less abundant in the root meristem zone and in stomatal precursor cells while it is enriched in root cap, trichoblasts, root hairs, leaf pavement cells, and trichomes. Additionally, we clearly observed ANN1 distribution patterns in inner root tissues such as cortex, endodermis, pericycle, and stele. The localization of free GFP as a negative control was examined in comparison to ANN1-GFP. Unlike ANN1-GFP which is localized in the cytoplasm and ER but not in the nucleus, free GFP accumulated in the nucleus because it is able to pass through nuclear pores.

Moreover, we were able to track spatio-temporal localization of ANN1-GFP with cellular and subcellular resolution. For dynamic processes such as cell division we used rather spinning disk microscopy, however with some limitations in spatial resolution. To overcome this problem, LSFM was used for long-term imaging of spatio-temporal localization of ANN1-GFP in *Arabidopsis* root at near-physiological conditions. LSFM revealed markedly high ANN1-GFP abundance in the cytoplasm of columella cells, however, a decreased amount of this protein was observed in lateral root cap cells. This is in agreement with results from *in situ* hybridization when *ANN1* expression was determined in the root cap, elongation zone, root hairs, and vascular tissue (Carroll et al., 1998; Clark et al., 2001; Clark et al., 2005b). Localization data could indicate AtANN1 involvement in the directed secretion of oligosaccharides by root cap cells (Clark et al., 2001) implicated in gravitropically-induced root bending. This was supported by the short root phenotype of a loss of function *ann1* T-DNA insertion mutant (Clark et al., 2005a; Clark et al., 2005b).

Our experiments confirm ANN1-GFP presence in secretory cells of the root cap, and in root hairs. Significantly stronger ANN1-GFP expression in trichoblast than in atrichoblast cells emphasizes the validity of the hypothesis about ANN1 involvement in polar growth of root hairs. Root hair development begins with the formation of a bulge at the basal part of a trichoblast followed by rapid tip growth (Mathur and Hülskamp, 2001). Typically, vesicles accumulate in the growing root hair tip and contain compounds of the plasma membrane and the cell wall such as xylans, xyloglucans, and pectins (Favery et al., 2001). Several early immunolocalization studies proposed involvement of annexins in secretory processes, based on their presence at the tips of polarly growing cells like pollen tubes and fern rhizoids (Blackbourn et al., 1992; Clark et al., 2005b), and association with the trans-Golgi membranes, Golgi-derived secretory vesicles and plasma membrane (Blackbourn and Battey, 1993). Apical zone accumulating secretory vesicles delivering cell wall components to the growing root hair tip is followed by the zone comprising most of the organelles. As the tip growth of root hairs terminates a vacuole extends toward the apex (Mathur and Hülskamp, 2001). We observed a net-like ANN1-GFP distribution resembling the endoplasmic reticulum (ER) structure in the cortex of differentiated root hairs. Colocalization of ANN1-GFP with ER was observed by Richter (2014) using ER marker in co-transformed tobacco leaves. These results suggested ANN1-GFP association with ER membranes including the nuclear envelope. In *Arabidopsis*, ANN1-GFP was not localized in the nucleus. Although plant annexins do not contain nuclear localization sequences, some of them may be imported to the nucleus by an unknown mechanism, as have been reported for crop species such as *Pisum sativum* (Clark et al., 1998), *Medicago sativa* (Kovács et al., 1998) and *Nicotiana tabacum* (Baucher et al., 2012).

Our analysis using lattice LSFM provided the experimental confirmation of ANN1-GFP localization near the nuclear envelope in elongating trichoblast. In addition, Airyscan CLSM revealed the accumulation of ANN1-GFP around spindle-shaped ER bodies and vesicular structures in hypocotyl cells.

Based on proteomic analyses, immunolocalization, radioactive labeling, and GFP reporter studies, ANN1 was identified and localized in the plasma membrane (PM, integral protein), ER, tonoplast, mitochondria, chloroplasts outer membrane, and cell wall (Laohavisit and Davies, 2011; Guelette et al., 2012). ANN1 as one of the lipid-binding proteins that can be cytoplasmic but has also been shown to bind membrane phospholipids, e.g. being involved in Ca^2+^ signaling as well as in callose formation (Andrawis et al., 1993; Mortimer et al., 2008). Using FM4-64 dye, we show osmotic stress-induced colocalization of ANN1-GFP with PM in the root epidermis. Because PM represents a key physiological interface barrier between plant cells and their surroundings, PM proteins including annexins might be important for plant stress response. Indeed, we report here a massive relocation and accumulation of ANN1-GFP at the PM and in Hechtian strands and reticulum in plasmolyzed cells after osmotic stress. Such mild plasmolysis caused by mannitol and NaCl was reversible, while deplasmolyzed cells still showed enrichment of ANN1-GFP at the PM showing a wavy pattern. These results suggest a possible osmoprotective role of ANN1-GFP during plasmolysis/deplasmolysis cycle. One possible explanation of osmotic stress-induced ANN1-GFP relocation to the PM is possible ion channel formation. Annexin is usually cytosolic under neutral pH in the cell. Osmotic stress promotes acidification of the cell, while a drop in pH might cause annexin hydrophobicity, relocation, and formation of oligomeric ion channel in the membrane (Gorecka et al., 2007). Other plausible explanation might be related to strongly enhanced membrane curvature in retracting protoplasts and osmotic stress-induced ANN1-GFP clustering and interaction with membrane anionic lipids involved in this process.

Our spatio-temporal LSFM observation of growing primary root revealed ANN1-GFP in mitotic figures of dividing cells. Co-immunolocalization study of ANN1-GFP and microtubules in the root tips provided additional support for ANN1 association with mitotic and cytokinetic microtubules such as spindles and phragmoplasts. Later ones are known to deliver cytokinetic vesicles to the cell plate during cytokinesis. One hypothesis suggests that annexins could activate different kinds of enzymes through interactions with them. Thus, annexins might regulate plasma membrane enzyme complexes such as callose and cellulose synthases (Verma and Hong, 2001; Hofmann et al., 2003). Callose is essential for cell plate formation during cytokinesis, during secondary cell wall synthesis in cotton ﬁbers and during pollen tube elongation. ANN1-GFP accumulated in the tip of growing root hair, when the cell wall is relaxed and must be accompanied by new cell wall synthesis to maintain the thickness. Among the other cell wall materials, mentioned above, primary cellulose layers are cell wall essential components. Cellulose is synthesized at the plasma membrane (Foreman and Dolan, 2001) that requires cellulose synthase activity. Beside expected functions, annexins have possible roles in vesicle-mediated secretion and endocytosis, also important for new cell wall formation (Dhonukshe et al., 2006). Further tests carried out by Proust et al. (1999) corroborate with our findings as identiﬁcation of two *Nicotiana* *tabacum* annexins (ANXD36 and ANXD37) that exhibited tissue-speciﬁc and cell cycle-dependent expression patterns. Annexin epitopes were localized to intercellular junctions forming a ring structure under the plasma membranes at the end of mitosis. The authors interpreted this pattern as a possible involvement of annexins in cell wall maturation. Hong and Verma (2013) and Shin and Brown (1999) proposed model of the callose synthase complex at the cell plate, according to which *Arabidopsis* CalS1 contains 16 putative transmembrane domains and utilize UDP-glucose transferase for substrate binding. They predict part of the complex may be annexins. Brown (2004) proposed that they might act as UDP-glucose transporters consequently affecting callose synthase activity by regulating substrate concentrations, because two cotton ﬁbers annexins were shown to bind UDP-glucose. Production of callose is important also in defense and wounding responses with certain annexins estimated to have roles in signaling.

The surface of annexin molecule is positively charged and can electrostatically interact with anionic membrane lipids such as phosphatidylserine or phosphoinositide (Laohavisit and Davies, 2011). This electrostatic lipid-protein interaction mechanism might be similar to other types of proteins such as BKI1, PINOID, or proteins containing the BAR domain and ALPS motif (Noack and Jaillais, 2020). In fact, annexins were proposed to bind membranes through their phospholipid-binding motif [GxGT-(38–40 residues)-D/E] (Konopka-Postupolska and Clark, 2017). Recently, plant annexins were found in membrane microdomain fractions as revealed by shot-gun proteomic approach (Gutierrez-Carbonell et al., 2016). Association of plant annexins with membrane lipid microdomains may function similarly to their animal counterparts (Konopka-Postupolska et al., 2011), which play roles in exo- and endocytosis and lipid microdomain signaling (Gutierrez-Carbonell et al., 2016). Moreover, annexins were identified as putative lipid-binding (e.g., phosphatidic acid and phosphatidylinositol phosphate) and transporting proteins in phloem exudate (Guelette et al., 2012). In this respect, we observed ANN1-GFP expression also in the root stele, which could be related to ANN1 role in phloem lipid transport. Consistently with this assumption, *in situ* hybridization revealed expression of *AtANN1* in *Arabidopsis* phloem (Clark et al., 2001), while immunolocalization of pea annexin P35 showed its accumulation in developing xylem and phloem (Clark et al., 1992).

In conclusion, this work provides new insight into complex developmental distribution and localization of ANN1-GFP in *Arabidopsis* seedlings, which can promote future genetic and functional annexin studies in plants.

## Data Availability Statement

The raw data supporting the conclusions of this article will be made available by the authors, without undue reservation. 

## Author Contributions

HR cloned the construct, transformed and selected transgenic *Arabidopsis* plants. MT acquired and made image post-acquisition analyses from the most of microscopy datasets with the help of OŠ and MO. NM obtained and deconvolved data from Lattice LSFM. MH made immunofluorescence labeling, performed microscopy imaging and image analysis. MT and PD performed protein extraction and immunoblotting. MT, JŠ, MO, and OŠ interpreted data and wrote the manuscript. JŠ provided infrastructure, partially supervised this research, and finalized manuscript. All authors contributed to the article and approved the submitted version.

## Funding

This work was supported by the ERDF project “Plants as a tool for sustainable global development” no. CZ.02.1.01/0.0/0.0/16_019/0000827 and by the student project IGA PrF_2018_031 of the Palacký University.

## Conflict of Interest

The authors declare that the research was conducted in the absence of any commercial or financial relationships that could be construed as a potential conflict of interest.
