# Supplementary material for: Advanced Microscopy Reveals Complex Developmental and Subcellular Localization Patterns of ANNEXIN 1 in Arabidopsis
Source: Front Plant Sci. 2020 Aug 5;11:1153. doi: 10.3389/fpls.2020.01153 (PMC7419693; doi:10.3389/fpls.2020.01153)
Supplement: Supplementary file 1 [file DataSheet_1.pdf]

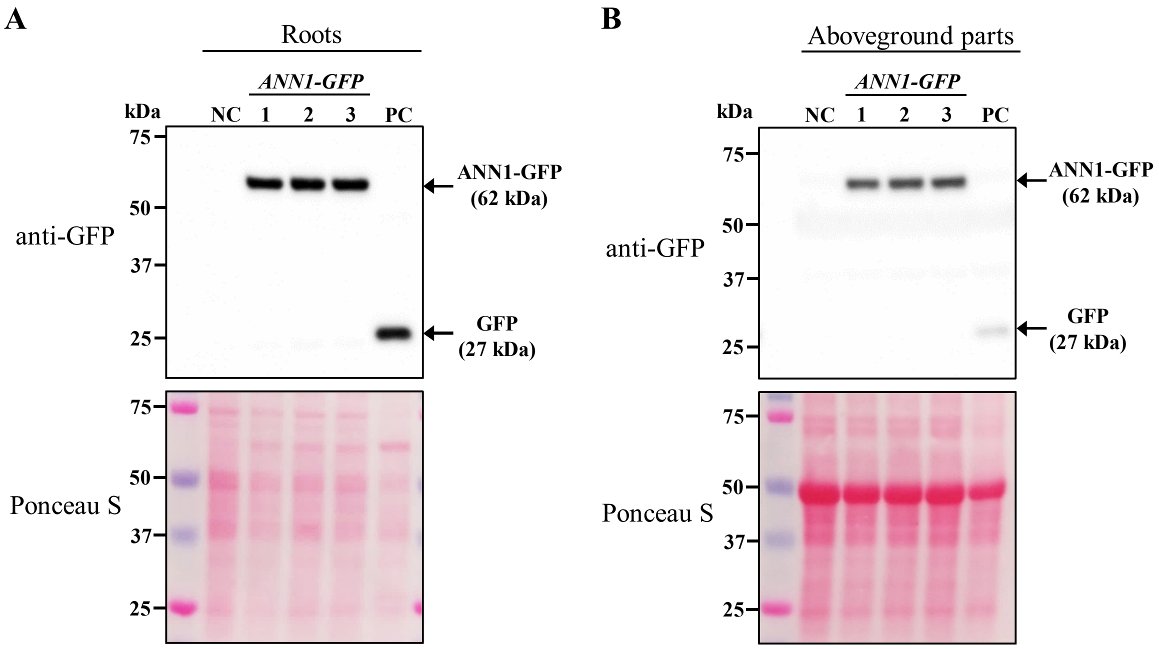

Supplementary Figure 1 Western blot detection of ANN1-GFP fusion protein using the anti-GFP antibody in **(A)** roots and **(B)** above-ground parts of *A. thaliana* Col-0 wild type (wt) and three independent lines stably transformed with *proANN1::ANN1:GFP* construct. Loading controls of proteins transferred on nitrocellulose membranes are visualized by Ponceau S. NC–negative control (wt); PC–positive control (free GFP produced in *A. thaliana* line carrying *35S::sGFP* construct).

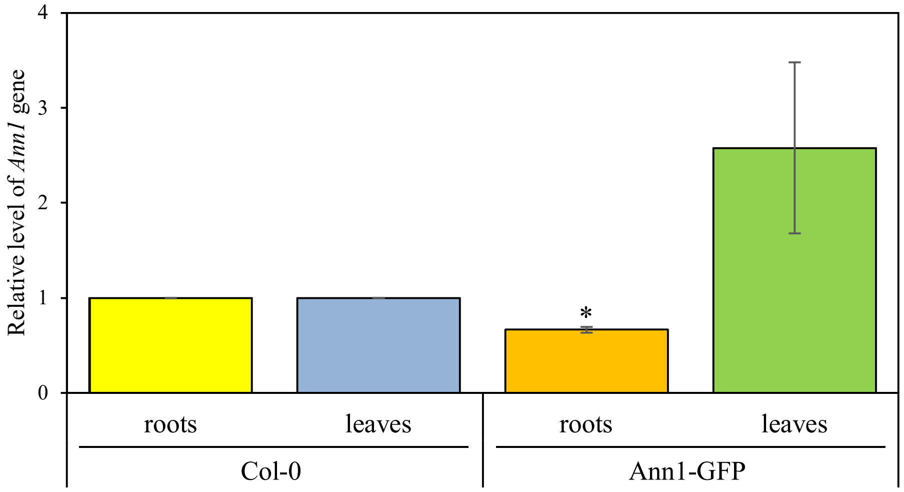

Supplementary Figure 2 Gene expression analysis of *ANN1* gene by quantitative real-time PCR (qRT-PCR) in roots and aerial parts of *A. thaliana* Col-0 wild type and line stably transformed with *proANN1::ANN1:GFP* construct. Statistical analysis was made in Microsoft Excel using Student's t-test ($n = 3$). *, $P \leq 0.05$ (as compared with Col-0). Error bars represent standard deviations.

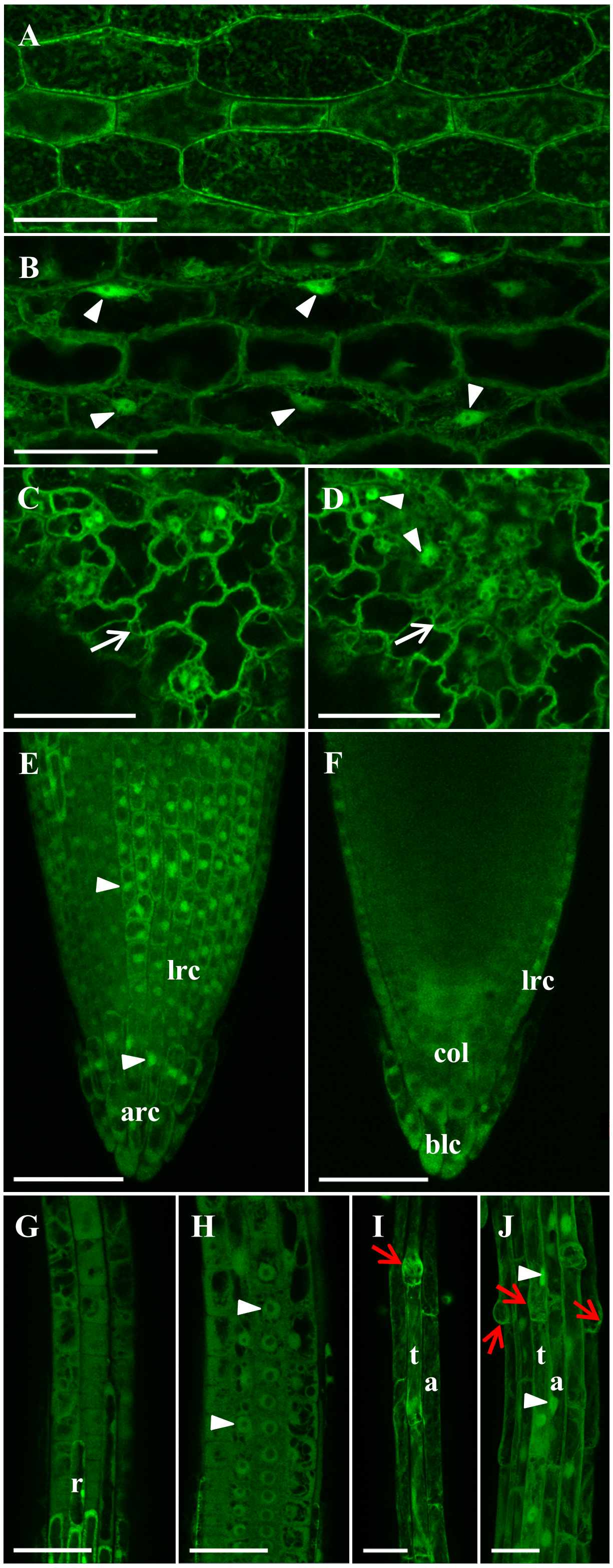

Supplementary Figure 3 Tissue- and cell-specific localization of free GFP in *A. thaliana* seedlings using CLSM. **(A)** Cortical zone of epidermal cells in hypocotyl and **(B)** their medial plane. **(C)** Pavement cells in cotyledon epidermis. White arrows indicate the cell with the surface area shown and in **(D)** its peripheral cytoplasm located proximally to the cotyledon. **(E)** The maximum intensity of the primary root tip and **(F)** medial Z-stack. lrc – lateral root cap cells, arc – apical root cap cells, col – columella cells, blc – border-like cells. **(G)** Meristematic zone, transition zone, and part of the elongation zone of the primary root, depicted is the most marginal Z-stack at the root surface. **(H)** The same zonation of the root from **(G)**. Z-stack shows the plane located adaxially to the root axis in the location of trichoblasts and atrichoblasts files. **(I)** Differentiation zone of the primary root. Maximum intensity of the first three root surface Z-stack layers captures cortexes of trichoblasts and atrichoblasts. **(J)** Maximum intensity projection of the primary root differentiation zone. a – atrichoblast, t – trichoblast, r – root cap cell, white arrowhead – nucleus, the red arrow indicates bulge of the future root hair. Scale bars = 50 μm.

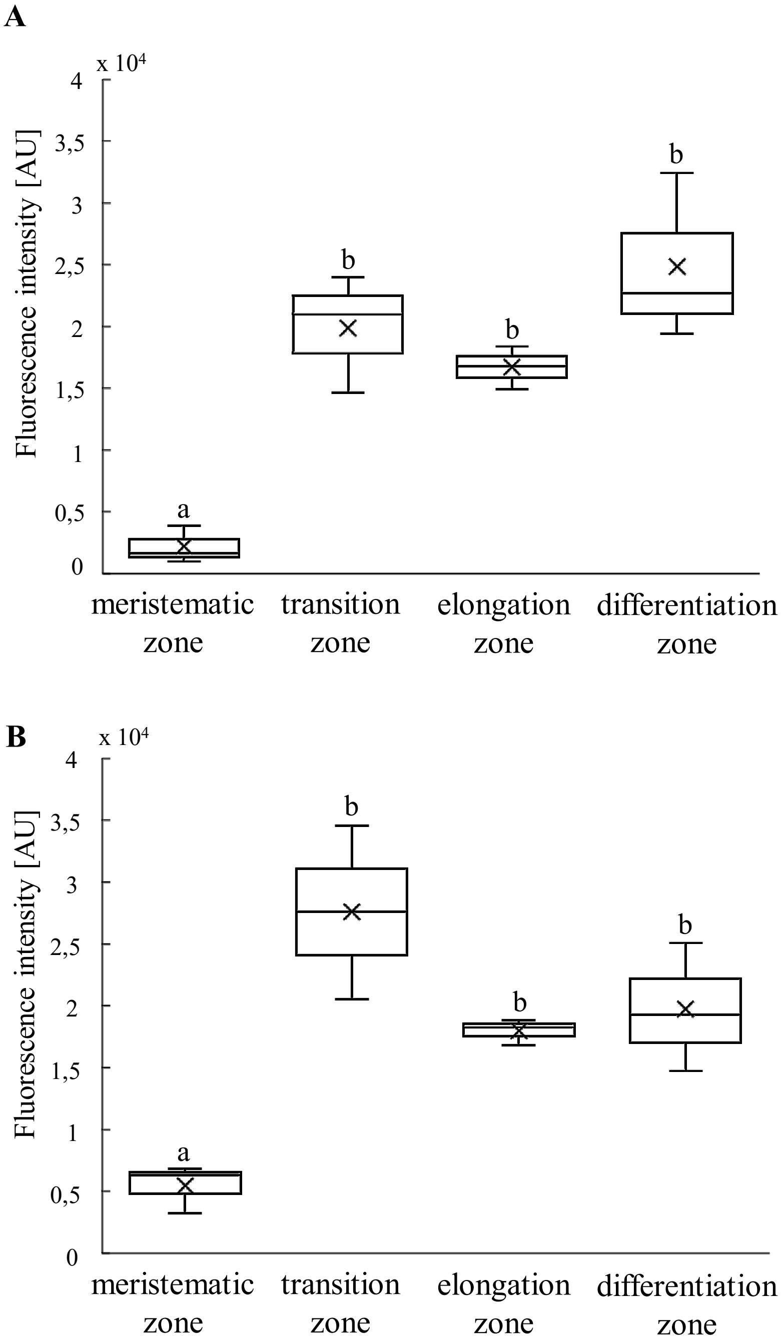

Supplementary Figure 4 Quantification of fluorescence intensity of ANN1-GFP distribution in individual zones of primary root during early *A. thaliana* development. Semi-quantitative analysis from data obtained using LSFM. **(A)** Fluorescence intensity of ANN1-GFP distribution along the longitudinal profiles from the medial plane and **(B)** along the horizontal profiles (cross-sections) of individual zones of the primary root. Profiles in both longitudinal **(A)** and horizontal **(B)** orientations spanned meristematic zone, transition zone elongation zone, and differentiation zone. Before the statistical analysis of images, a uniform correction of brightness and contrast was done. Statistic was calculated by one-way ANOVA using Holm's method ($n = 3$). Different lower-case letters indicate a significant difference at the levels $P \leq 0.05$.

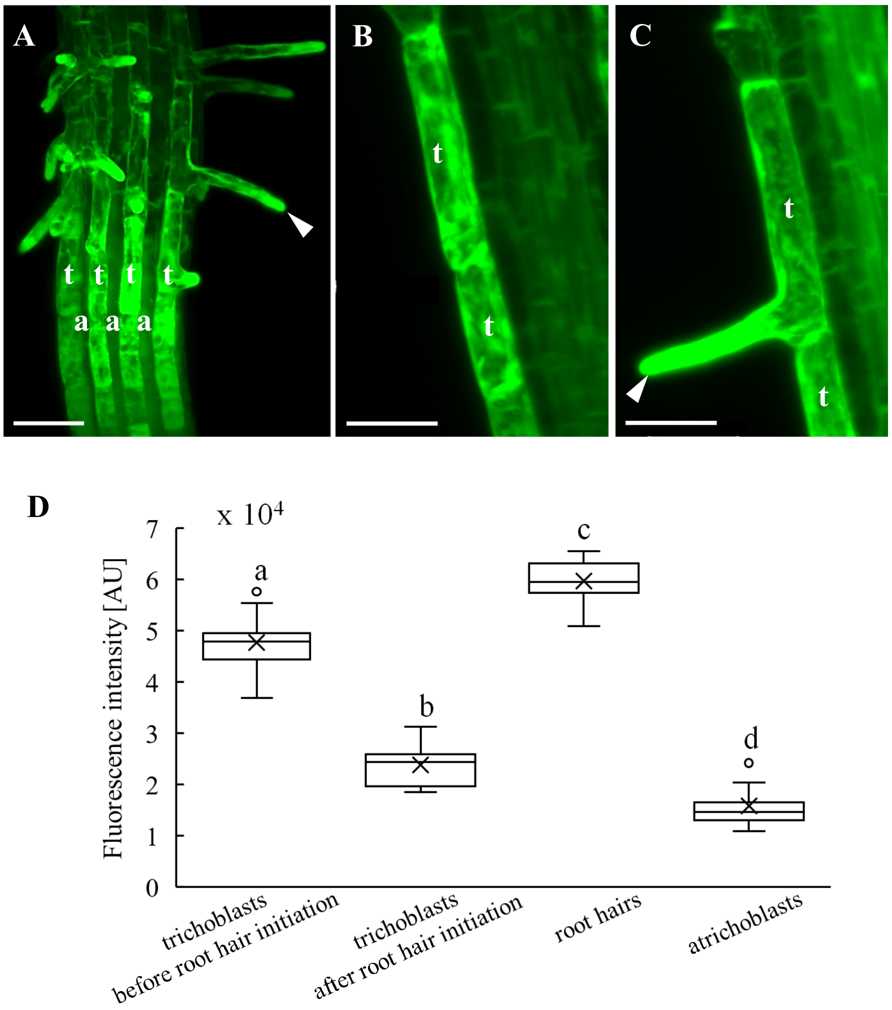

Supplementary Figure 5 Localization of ANN1-GFP in the epidermis of *A. thaliana* primary root stably expressing *proANN1::ANN1:GFP* construct using LSFM. **(A)** Differences in cell-type-specific production of ANN1-GFP in trichoblast (t) and atrichoblast (a) cell files within the root differentiation zone. **(B)** A high amount of evenly distributed ANN1-GFP in trichoblasts before root hair initiation. **(C)** Relocation of ANN1-GFP from the cytoplasm of the trichoblasts towards growing root hair (arrowhead). **(D)** Quantification of fluorescence intensity of ANN1-GFP in trichoblasts before root hair initiation, in trichoblasts after root hair initiation, in growing root hairs, and atrichoblasts. Fluorescence intensity of ANN1-GFP was measured along three different planes of the maximum intensity projection from individual optical sections of three independent respective cell types and in three different plants. In statistically evaluated images a uniform correction of brightness and contrast was done before the export for the analysis. Statistic was calculated by one-way ANOVA using Holm's method ($n = 9$). Different lower-case letters indicate a significant difference at the level $P \leq 0.01$. Scale bars = 50 µm.

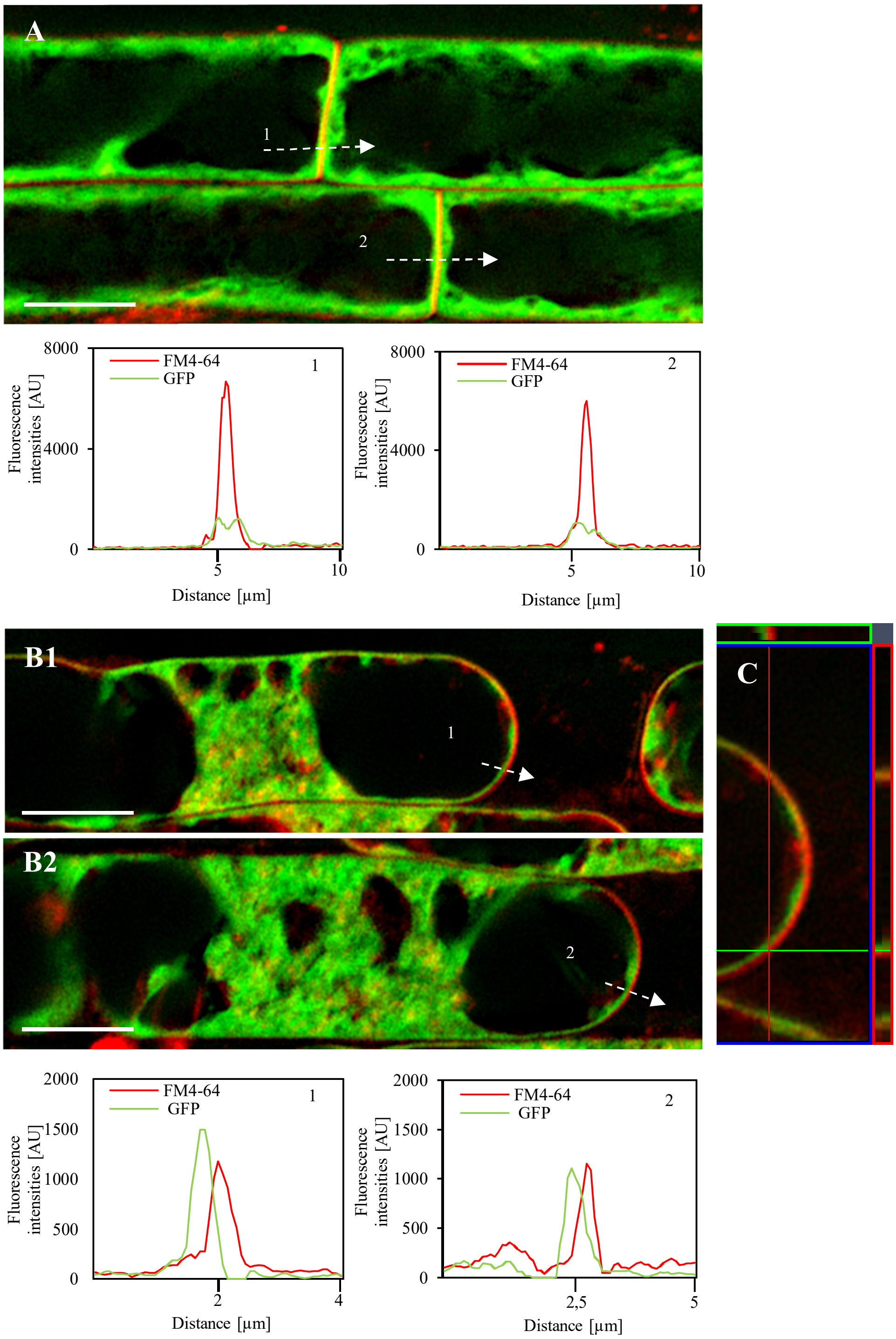

Supplementary Figure 6 Localization of free GFP in plasmolyzed root epidermal cells after salt stress as revealed by high-resolution Airyscan CLSM. **(A)** Root epidermal cells in the elongation root zone carrying free GFP and co-labeled with FM4-64 (red) for detection of the plasma membrane under control conditions. Fluorescence intensity profiles measured along dotted white arrows 1 and 2 in **(A)** showing distinct separated free GFP and FM4-64 peaks, suggesting no obvious colocalization. **(B)** Salt stress-induced plasmolysis in root epidermal cells carrying free GFP with FM4-64 labeled plasma membranes in the elongation root zone. Fluorescence intensity profiles measured along dotted white arrows 1 and 2 in **(B)** showing no overlap of FM4-64 and free GFP peaks, suggesting no colocalization. **(C)** Orthogonal view of plasmolyzed root epidermal cell carrying free GFP and co-labeled with FM4-64 showing clearly separated green and red signals. Scale bars = 10 µm.

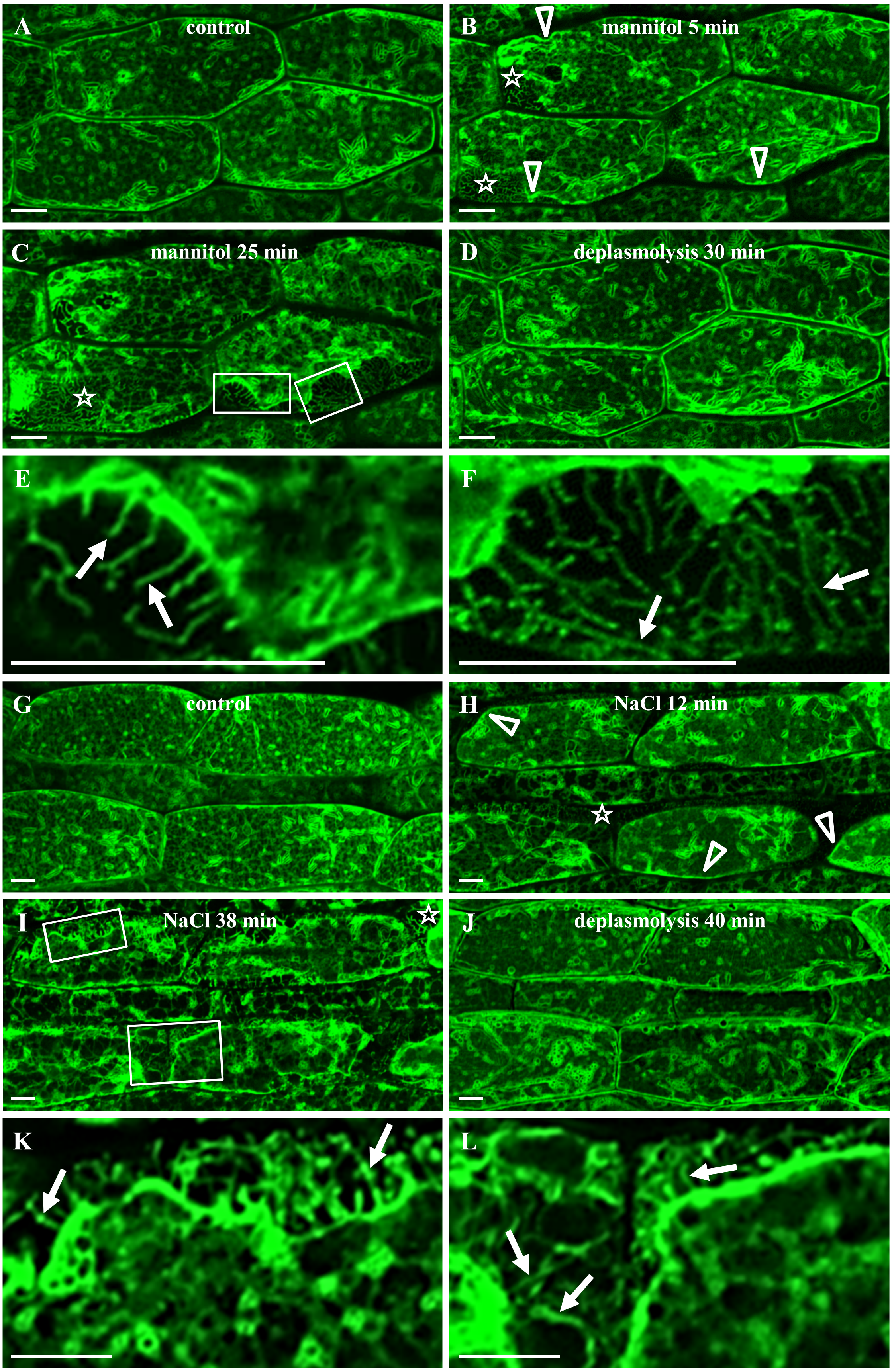

A
control
B
mannitol 5 min
C
mannitol 25 min
D
deplasmolysis 30 min
E
F
G
control
H
NaCl 12 min
I
NaCl 38 min
J
deplasmolysis 40 min
K
L

Figure S7

Supplementary Figure 7 Plasmolysis and following deplasmolysis of hypocotyl cells treated with non-ionic **(A–F)** and ionic **(G–L)** osmotics. Note the intracellular accumulation of ANN1-GFP around spindle-shaped ER bodies and vesicular structures. **(A)** Localization of ANN1-GFP in control conditions and **(B,C)** relocation and accumulation of ANN1-GFP on retracting portions of PM (arrowheads in **B**) and Hechtian reticulum (stars) after plasmolysis with 1M mannitol for indicated time points. **(D)** Recovery of retracted protoplasts after deplasmolysis following mannitol treatment. Note the persistent accumulation of ANN1-GFP at the PM and disappearance of Hechtian strands and reticulum. **(E,F)** Detailed views on areas indicated by boxes in **(C)** showing Hechtian strands (arrows) and Hechtian reticulum. **(G)** Localization of ANN1-GFP in control conditions and **(H,I)** relocation and accumulation of ANN1-GFP on retracting portions of PM (arrowheads in **H**) and Hechtian reticulum (stars) after plasmolysis with 500 mM NaCl for indicated time points. **(J)** Recovery of retracted protoplasts after deplasmolysis following NaCl treatment. Note the persistent accumulation of ANN1-GFP at the PM showing wavy pattern and disappearance of Hechtian strands and reticulum. **(K,L)** Detailed views on areas indicated by boxes in **(I)** showing Hechtian strands (arrows) and Hechtian reticulum. Scale bars = 10 μm.

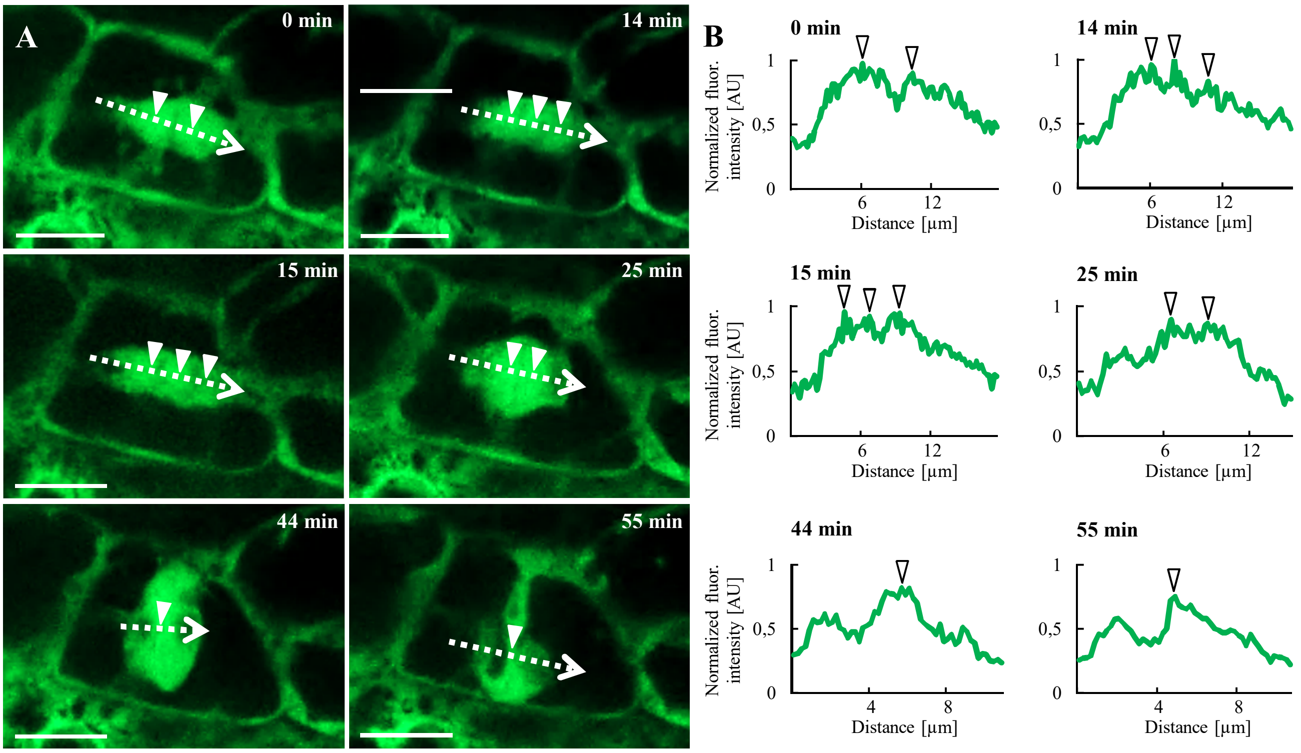

Supplementary Figure 8 Time-lapse *in vivo* imaging of subcellular localization of ANN1-GFP during cell division in petiole epidermal cell of *A. thaliana* stably expressing *proANN1::ANN1:GFP* construct using spinning disk microscopy. **(A)** ANN1-GFP decorating mitotic spindle (**0–15 min**), early phragmoplast (**15–25 min**), and late phragmoplast (**44–55 min**). **(B)** Fluorescence intensity profiles of ANN1-GFP distribution measured along the indicated dotted arrows in respective cell division stages. Arrowheads (white and black) indicate a maximum of measured fluorescence intensities. Scale bars = 20 µm.

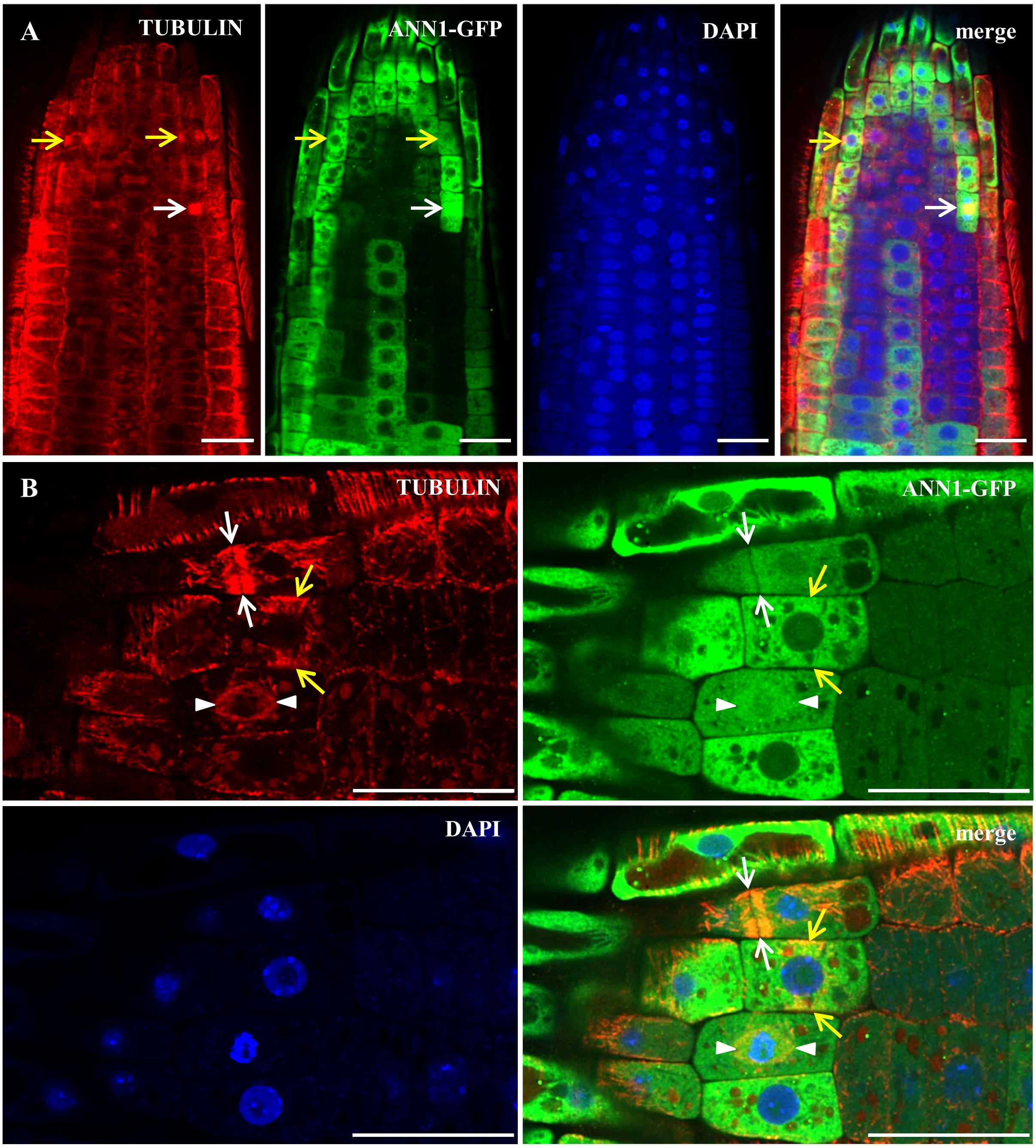

Supplementary Figure 9 Whole-mount immunofluorescence localization of tubulin and ANN1-GFP in *A. thaliana* primary root stably expressing *proANN1::ANN1:GFP* construct using Airyscan CLSM. **(A)** Overview of the primary root tip and **(B)** detailed view of lateral root cap cells. Microtubules are presented in red, ANN1-GFP is presented in green, nuclei stained with DAPI are presented in blue, merge – merged all channels. Yellow arrows point to pre-prophase bands, arrowheads point to mitotic spindles, and white arrows point to phragmoplasts. Scale bars = 20 µm.

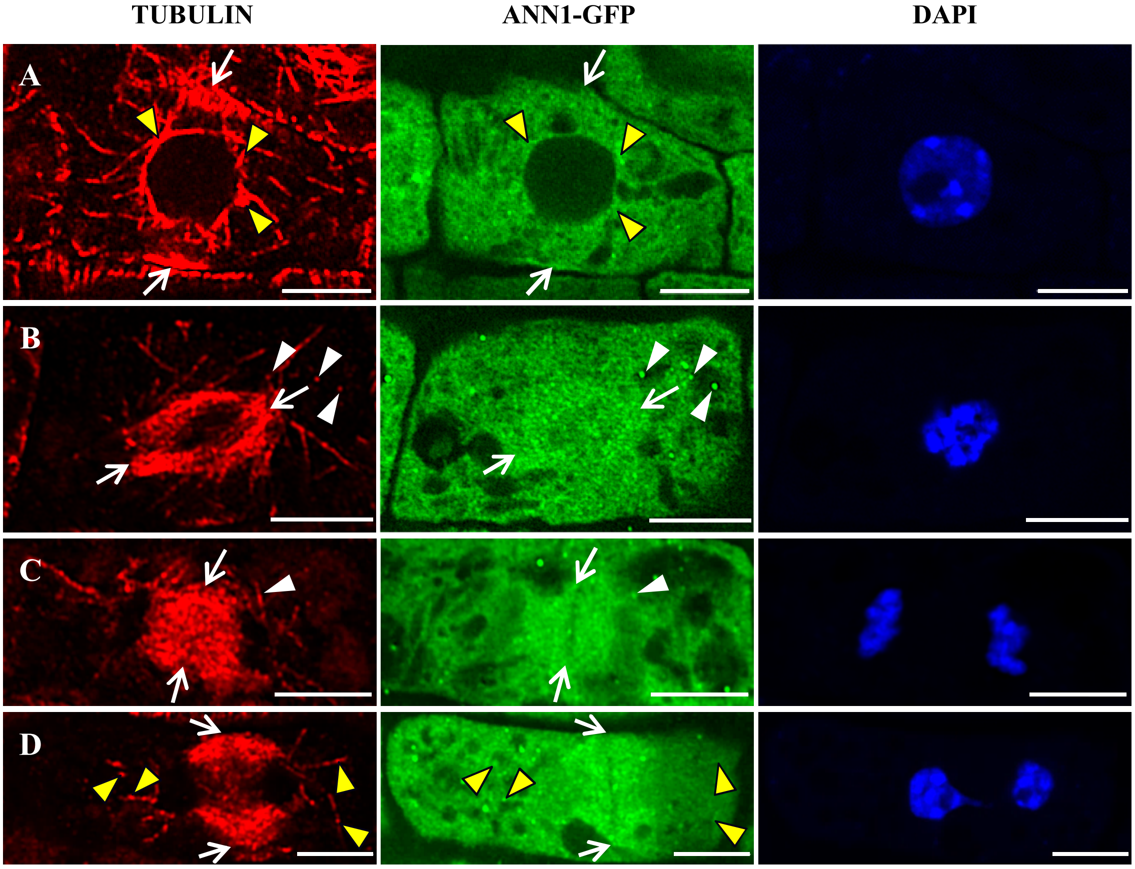

Supplementary Figure 10 Whole-mount immunofluorescence colocalization of microtubules and ANN1-GFP in fixed lateral root cap cells during cell division using Airyscan CLSM. Individual division stages (indicated by white arrows) are depicted according to the morphology of nuclei stained with DAPI. Microtubules form **(A)** pre-prophase band, **(B)** mitotic spindle, **(C)** early phragmoplast, and **(D)** late phragmoplast. Microtubules are shown in red, ANN1-GFP in green, and DNA stained with DAPI in blue. Yellow arrowheads indicate colocalization of ANN1-GFP with filamentous microtubules, white arrowheads point to colocalization of ANN1-GFP with tubulin in a spot like-manner. Scale bars = 5 µm.
